# Leader-Containing Uncapped Viral Transcript Activates RIG-I in Antiviral Stress Granules

**DOI:** 10.1371/journal.ppat.1005444

**Published:** 2016-02-10

**Authors:** Seong-Wook Oh, Koji Onomoto, Mai Wakimoto, Kazuhide Onoguchi, Fumiyoshi Ishidate, Takahiro Fujiwara, Mitsutoshi Yoneyama, Hiroki Kato, Takashi Fujita

**Affiliations:** 1 Laboratory of Molecular Genetics, Institute for Virus Research, Kyoto University, Kyoto, Japan; 2 Division of Molecular Immunology, Medical Mycology Research Center, Chiba University, Chiba, Japan; 3 Laboratory of Molecular and Cellular Immunology, Graduate School of Biostudies, Kyoto University, Kyoto, Japan; 4 Department of Microbiology and Immunology, University of California, San Francisco, California, United States of America; 5 Carl Zeiss MicroImaging Co., Ltd., Tokyo, Japan; 6 Center for Meso-Bio Single-Molecule Imaging (CeMI), Institute for Integrated Cell-Material Sciences (iCeMS), Kyoto University, Kyoto, Japan; Institute for Virology, GERMANY

## Abstract

RIG-I triggers antiviral responses by recognizing viral RNA (vRNA) in the cytoplasm. However, the spatio-temporal dynamics of vRNA sensing and signal transduction remain elusive. We investigated the time course of events in cells infected with Newcastle disease virus (NDV), a non-segmented negative-strand RNA virus. RIG-I was recruited to viral replication complexes (vRC) and triggered minimal primary type I interferon (IFN) production. RIG-I subsequently localized to antiviral stress granules (avSG) induced after vRC formation. The inhibition of avSG attenuated secondary IFN production, suggesting avSG as a platform for efficient vRNA detection. avSG selectively captured positive-strand vRNA, and poly(A)^+^ RNA induced IFN production. Further investigations suggested that uncapped vRNA derived from read-through transcription was sensed by RIG-I in avSG. These results highlight how viral infections stimulate host stress responses, thereby selectively recruiting uncapped vRNA to avSG, in which RIG-I and other components cooperate in an efficient antiviral program.

## Author Summary

RIG-I plays a critical role in sensing cytoplasmic vRNA and triggering a downstream signaling cascade to produce the antiviral cytokine IFN. Over the past decade, a number of *in vitro* studies have been undertaken to address the nature of the ligands for RIG-I, and it was demonstrated that RNA species forming a 5’-triphosphate and short double-stranded motif preferentially activates RIG-I. The next challenge to address would be understanding the physiological behavior of RIG-I in the context of authentic virus infection. In this study, by monitoring the time-course events in NDV-infected cells, we demonstrated that RIG-I senses vRNAs that accumulate in vRC and subsequently in host avSG. We discovered that avSG contains vRNA derived from read-through transcription, which acts as a novel RIG-I ligand. Our findings illustrated how RIG-I encounters its natural ligands, and initiates and integrates IFN signaling through the course of virus infection.

## Introduction

Retinoic acid-inducible gene I (RIG-I), a DExD/H-box RNA helicase family protein, is a crucial cytosolic viral RNA (vRNA) sensor that initiates signal transduction to produce antiviral cytokines, namely, type I and III interferons (IFN-α/β and IFN-λ) [1–4]. RIG-I selectively recognizes relatively short double-stranded RNA (<100 nt), and this recognition is markedly enhanced by the presence of 5’-triphosphate [5–8]. Once RIG-I is activated, it physically associates with downstream IFN-β promoter stimulator 1 (IPS-1, also termed MAVS, VISA, or Cardif), which is anchored to the mitochondrial outer membrane [9–12]. The RIG-I/IPS-1 interaction leads to the recruitment of a variety of signaling adaptors in order to activate transcription factors including IFN regulatory factors (IRF) 3 and 7 as well as NF-κB. These transcription factors activate the genes coding for cytokines and IFNs in addition to IFN-stimulated genes (ISG).

Viral infection is a pivotal stressor that stimulates cells to execute intrinsic anti-stress strategies. As a part of the stress response, cells provisionally interrupt translational machinery in order to avoid excrescent proteins and shelter accumulated mRNA by sequestering them in cytoplasmic protein complexes, termed stress granules (SG) [13]. SG are typically composed of 40S ribosomal subunits, a subset of translation initiation factors (eIF2, 2B, 3, 4A, 4B, 4E and 4G), and host RNA-binding proteins such as Ras GTPase-activating protein-binding protein (G3BP), poly(A)-binding protein (PABP), human antigen R (HuR), T-cell intracellular antigen-1 (TIA-1), and its related protein TIAR [13–17]. A variety of viruses have been shown to induce the formation of SG in infected cells [18], which is strictly regulated in a steady state; however, once cells are exposed to viral infections, double-stranded RNA (dsRNA)-inducible protein kinase R (PKR) is activated by viral dsRNA (vdsRNA), an intermediate product generated within the viral replicative life cycle [19]. Activated PKR further phosphorylates the eIF2 α-subunit (eIF2α) and then recruits SG components to form the complex [13].

We recently reported that SG played a positive role in antiviral IFN signaling against influenza A virus (IAV) lacking an IFN-inhibitory NS1 protein (IAVΔNS1) by recruiting RIG-I and a set of antiviral host proteins to detect the viral infection [20]. Moreover, another DExD/H-box RNA helicase protein DHX36 acts with PKR to induce SG, hence facilitates detection of vRNA by RIG-I [21]. These findings shed light on the function of SG as antiviral SG (avSG), a substantial platform for IFN-inducing signaling triggered by RIG-I. However, vRNA species in avSG have not yet been characterized. Few studies have interpreted the spatial and temporal behaviors of RIG-I in virus-infected cells in relation to the induction of IFN.

Newcastle disease virus (NDV) of *Paramyxoviridae*, a negative-single strand RNA virus, has been shown to efficiently trigger the production of IFN in human and rodent cells. NDV encodes a nucleocapsid protein (N), phosphoprotein (P), matrix protein (M), fusion protein (F), hemagglutinin-neuraminidase protein (HN), and large RNA-dependent RNA polymerase protein (L). These genes are arranged on non-segmented viral genomic RNA (vgRNA) with extracistronic sequences, known as leader (Le) and trailer (Tr), in an order corresponding to 3’-Le-N-P-M-F-HN-L-Tr-5’ [22]. The viral polymerase first transcribes Le sequence into positive-strand RNA without terminal modification [23]. After Le transcription, the polymerase re-initiates transcription for N gene to yield N mRNA with 5’-m7G cap and 3’-poly(A) tail. Similarly, the polymerase synthesizes other viral mRNAs (vmRNAs) by transcribing downstream genes without dissociation from the template. In later stages of the infection, polymerase switches to the replication mode in order to synthesize the entire length of viral antigenomic RNA as a template for the synthesis of vgRNA. The general strategies of transcription and replication are similar across negative-single strand RNA viruses [24], and the vRNA species produced during the replication cycle are sensed by RIG-I-like receptors [25,26].

In the present study, we analyzed stress responses in NDV-infected cells in relation to the induction of IFN gene activation. We found that NDV infection induced viral replication complexes (vRC) to which RIG-I was recruited. The appearance of vRC was followed by the formation of avSG, which also recruited RIG-I. The inhibition of avSG diminished signaling for the IFN induction upon NDV infection, suggesting that avSG played an important role in signal amplification. Our results also indicated that poly(A)-containing vmRNAs migrated from vRC to avSG, and that Le-N fusion RNA activated RIG-I in avSG. The results of the present study demonstrated the spatio-temporal dynamics of vRNA detection in triggering the induction of IFN.

## Results

### NDV infection induced vRC and avSG

**Fig 1:**
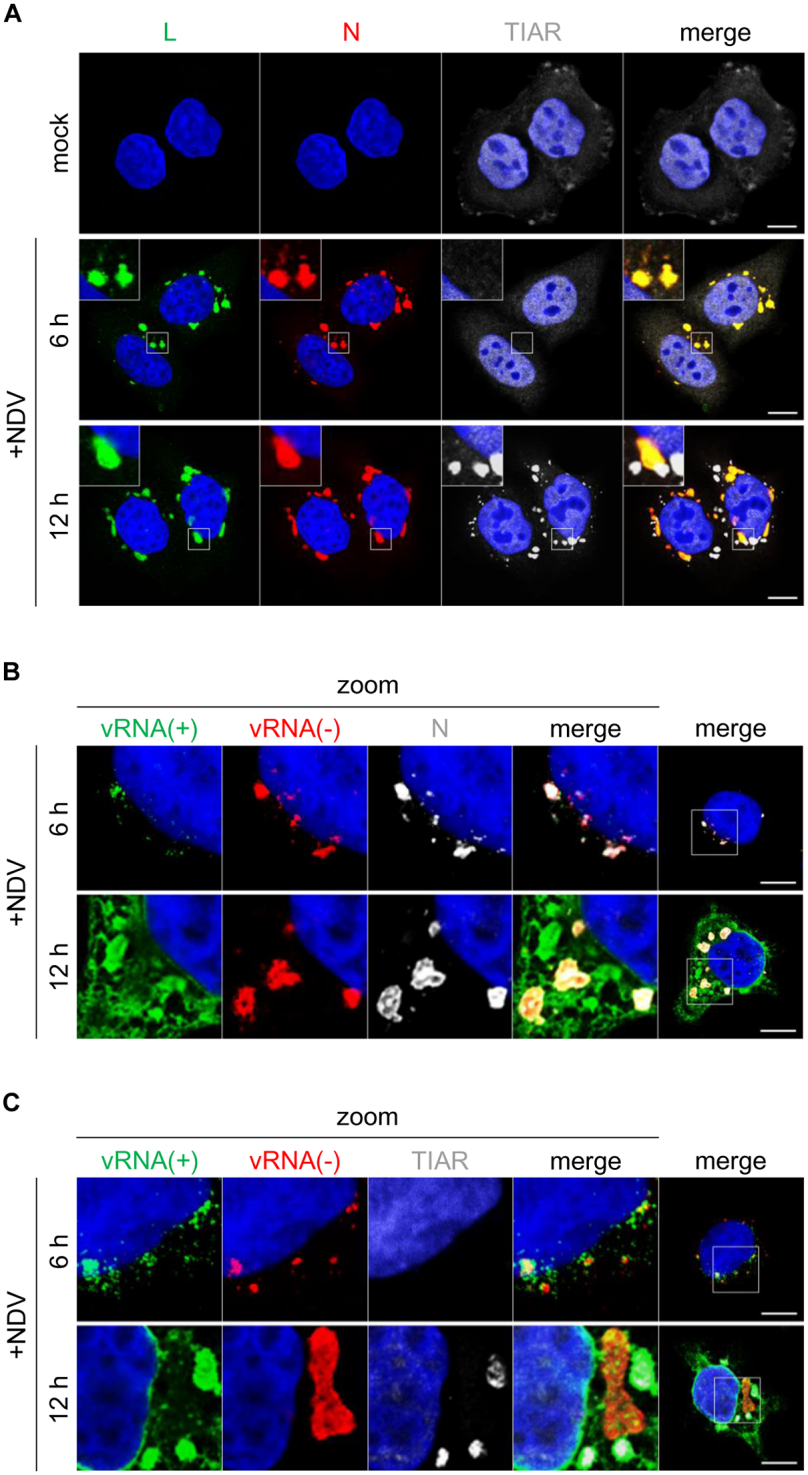
NDV infection induced formation of vRC independently of host avSG and vRNA(+) migrated from vRC to SG. **(A)** Confocal micrographs of NDV-infected cells. HeLa cells were either mock treated or infected with NDV (MOI = 1) for 6 and 12 hours and immunostained for L (green), N (red), and TIAR (white). Nuclei were stained with DAPI (blue). The boxed area was enlarged and displayed on the upper left of the image. The white scale bar corresponds to 10 μm. **(B and C)** Distribution of NDV vRNAs in 6-h and 12-h NDV-infected (MOI = 1) HeLa cells. NDV vRNA(-) (red) and vRNA(+) (green) were detected by the RNA-FISH method. N (B) and TIAR (C) were immunostained with their respective antibodies (white). Nuclei were stained with DAPI (blue). A merged image at an original magnification was shown in the rightmost panel. The white scale bar corresponds to 10 μm. See also S1, S2 and S3 Figs.

We examined viral replication and cellular stress responses by immunostaining NDV-infected HeLa cells. Viral replication was detected by the accumulation of viral N and L proteins (Fig 1A). These viral proteins localized in granules 6 hr post-infection (hpi) and persisted to 12 hpi. We detected vRNA by RNA-FISH, which allowed us to specifically detect vRNA with undetectable background signal in mock-infected cells (S1 Fig). The results revealed that these granules also contained positive-strand and negative-strand vRNA (vRNA(+) and vRNA(-)) (Fig 1B). Therefore, we refer these as viral replication complexes (vRC). In order to monitor host stress responses, we detected avSG using antibody to TIAR (Fig 1A and 1C). We also tested other avSG markers, TIA-1, G3BP1, and eIF3η (S2 and S3 Figs) and confirmed that all of these markers detect avSG. avSG were not detected at 6 hpi, by which time vRC had already been identified; however, avSG were clearly observed as distinct granules from vRC at 12 hpi (Fig 1A and S3 Fig). avSG and vRC did not completely merge, except for occasional contact between vRC and avSG (below). avSG coincided with vRNA(+), but not with vRNA(-) (Fig 1C). These results suggested that vRNA(+) was generated in vRC and then translocated into avSG.

Certain types of RNA viruses including NDV have been shown to produce vdsRNA [27–31]. Therefore, we examined subcellular localization of vdsRNA by immunostaining with an anti-dsRNA antibody, which detected >40 bp dsRNA [32]. We demonstrated that vdsRNA was not detectable within vRC at 6 hpi, but was clearly present at 12 hpi (S4 Fig). The detection of vdsRNA was restricted to vRC which contains L and vRNA(-). avSG contains vRNA(+) and TIAR but apparently devoid of vdsRNA. We confirmed specificity of anti dsRNA antibody through the loss of reactivity after ribonuclease digestion (RNase A and III) (S5 Fig).

### Relationship between vRC formation and early *IFNB* gene activation

**Fig 2:**
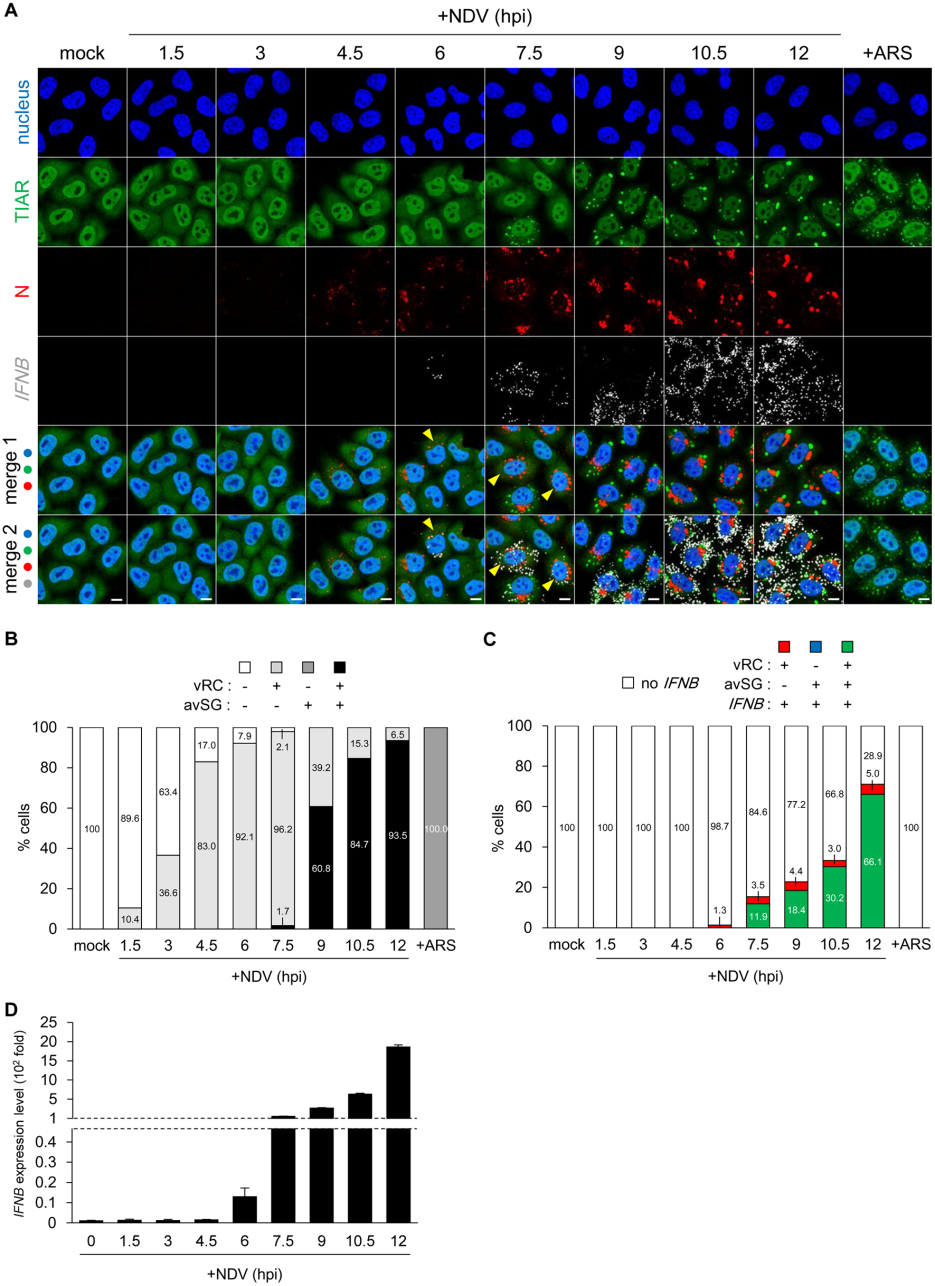
Formation of NDV vRC correlated with primary induction of *IFNB* mRNA. **(A)** Time course of vRC, avSG, and *IFNB* mRNA in NDV-infected cells. HeLa cells were infected with NDV (MOI = 1) for the indicated time or treated with 0.5 mM sodium arsenite (ARS) for 30 minutes. After fixation, the cells were immunostained for TIAR (green) and N (red). *IFNB* mRNA (white) was detected by the RNA-FISH method. Nuclei were stained with DAPI (blue). Merge 1: nucleus, TIAR, N, merge 2: nucleus, TIAR, N and *IFNB* mRNA. The yellow arrowheads are cells double-positive for vRC and *IFNB* mRNA (without avSG). The white scale bar corresponds to 10 μm. **(B and C)** Quantitative analysis of vRC, avSG, and *IFNB* mRNA expression. Approximately 300 cells at the indicated time points of NDV infection (MOI = 1) or ARS treatment were counted by MetaMorph software. Cells were categorized according to the existence of vRC, avSG, and *IFNB* mRNA as shown on the top. Percentages were indicated inside the data bar. **(D)** HeLa cells were infected with NDV (MOI = 1) for the indicated time up to 12 hours. Expression levels of *IFNB* mRNA were measured by RT-qPCR. Data are represented as means ±SD.

In order to elucidate the biological significance of vRC and avSG in antiviral innate immunity, we examined the kinetics of the appearance of these granules along with *IFNB* gene expression at every 1.5 hours up to 12 hpi (Fig 2A). Our RNA-FISH detection of *IFNB* transcript correlated with the nuclear translocation of IRF-3 and NF-κB (S6 Fig). vRC (N) were initially detected as small granules (4.5 hpi), the size of which subsequently increased (7.5 hpi). On the other hand, avSG (TIAR) were detected as late as 7.5 hpi and persisted thereafter. Quantification (Fig 2B) revealed the temporal appearance of these granules: vRC-positive cells (light gray) were first detected at 1.5 hpi, and reached >90% at 6 hpi; avSG-positive cells (black) were detected at 7.5 hpi and reached > 90% at 12 hpi. No cells without vRC exhibited avSG, except for those treated with arsenite (dark gray), which was used as a positive control for the formation of SG. Quantification of *IFNB* FISH (Fig 2C) revealed that *IFNB* mRNA-positive cells (red + green) were first detected at 6 hpi and their population subsequently increased. At 6 hpi, *IFNB* mRNA-positive cells were all vRC-positive (red). Cells triple positive for vRC, avSG, and *IFNB* mRNA (green) were detected after 7.5 hpi, and this population increased to 66.1% at 12 hpi, suggesting that this type of cell was the main cell producing IFN-β. Cells positive for vRC and *IFNB* mRNA, but negative for avSG (red) remained at approximately 3–5%. We compared RNA-FISH results with RT-qPCR (Fig 2D). The results obtained by two distinct methods are closely correlated and confirmed that gene expression was first detected at 6 hpi and markedly increased up to 12 hpi. Since *IFNB* mRNA was detected in cells exhibiting vRC at 6 hpi, we speculated that the formation of vRC was responsible for *IFNB* gene expression at 6 hpi (Fig 2B). However, a population of cells triple positive for vRC, avSG, and *IFNB* mRNA (green) markedly increased, suggesting that avSG contributed to enhancing *IFNB* gene expression.

### Localization of RIG-I in vRC and avSG

**Fig 3:**
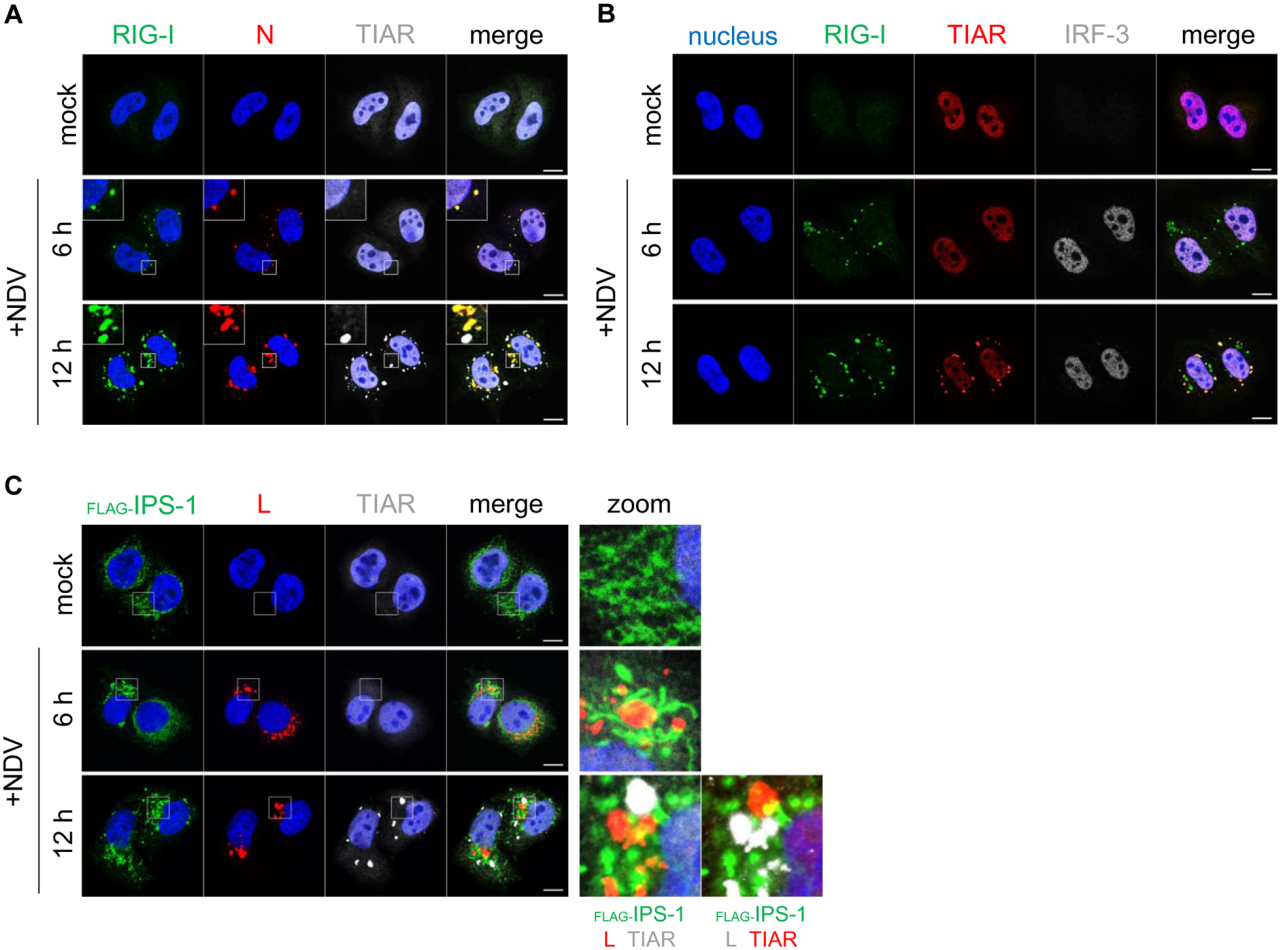
RIG-I localized in NDV vRC and avSG. **(A and B)** HeLa cells were either mock treated or infected with NDV (MOI = 1) for 6 and 12 hours. Cells were immunostained for RIG-I (green), N (red), and TIAR (white) (A), or RIG-I (green), TIAR (red), and IRF-3 (white) (B). **(C)** FLAG-IPS-1/HeLa cells were either mock treated or infected with NDV (MOI = 1) for 6 and 12 hours. The cells were immunostained for FLAG (green), L (red), and TIAR (white). Nuclei were stained with DAPI (blue). The white scale bar corresponds to 10 μm. The boxed area was enlarged and displayed on the right (zoom). Partial co-localization between IPS-1 and L (vRC) or TIAR (avSG) is shown by displaying them in green and red, respectively (zoom).

Since NDV infection is detected by RIG-I, we examined its localization in NDV-infected cells (Fig 3A). RIG-I co-localized with vRC at 6 hpi when avSG was not induced. At 12 hpi, RIG-I co-localized with vRC and avSG. These results were consistent with the formation of vRC and avSG coinciding with the accumulation of *IFNB* mRNA (Fig 2) and nuclear translocation of IRF-3 (Fig 3B). RIG-I transduces a signal to an adaptor, IPS-1, which is expressed on mitochondria. We monitored the localization of IPS-1 using HeLa cells stably expressing FLAG-tagged IPS-1 [33]. At 6 hpi, IPS-1 localized in the proximity of vRC, showing a yellow color in the periphery of vRC (as probed by anti-L), suggesting their close interaction (Fig 3C). At 12 hpi, avSG were clearly detected and partially co-localized with IPS-1 (Fig 3C, bottom right). We confirmed that endogenous IPS-1 re-localized upon NDV infection (S7 Fig) and interacted with vRC and avSG (S8 Fig).

### avSG formation was required for full activation of *IFNB* gene expression

**Fig 4:**
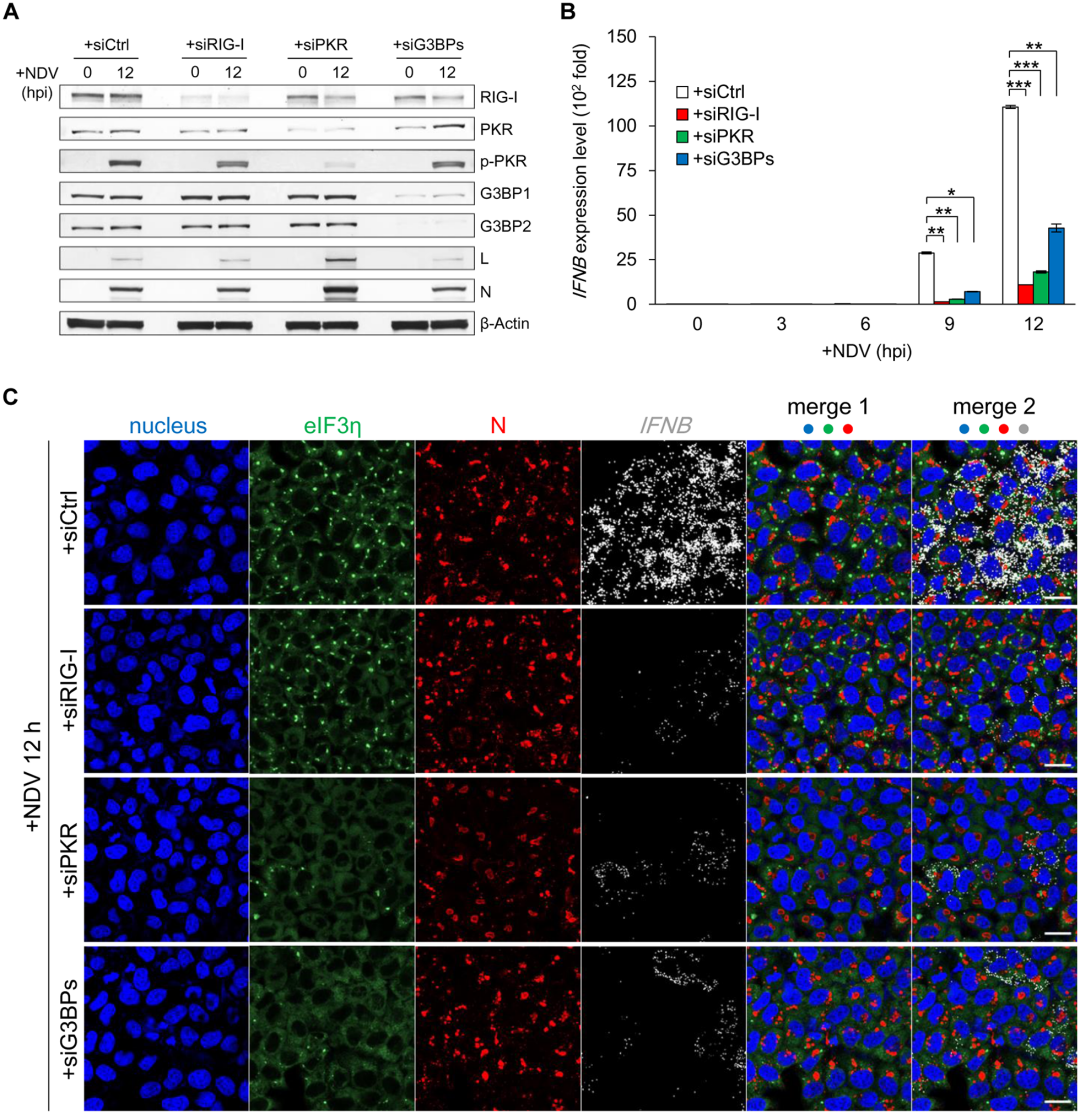
Inhibition of avSG formation led to diminished *IFNB* gene expression. **(A-C)** HeLa cells were transfected with siRNAs; siCtrl, siRIG-I, siPKR, or siG3BPs (for G3BP1 and G3BP2). After transfection, the cells were infected with NDV (MOI = 1) for the indicated time. (A) Expression levels of the indicated proteins were analyzed by western blotting. (B) *IFNB* mRNA expression was analyzed by RT-qPCR. Data are represented as means ±SD (t-test: ***p<0.01, **p<0.05, *p<0.1, NS = not significant). (C) Cells were immunostained for eIF3η (green) and N (red). *IFNB* mRNA (white) was detected by the RNA-FISH method. Nuclei were stained with DAPI (blue). Merge 1: nuclei, eIF3η, N. Merge 2: nuclei, eIF3η, N, and *IFNB* mRNA. The white scale bar corresponds to 20 μm.

**Fig 5:**
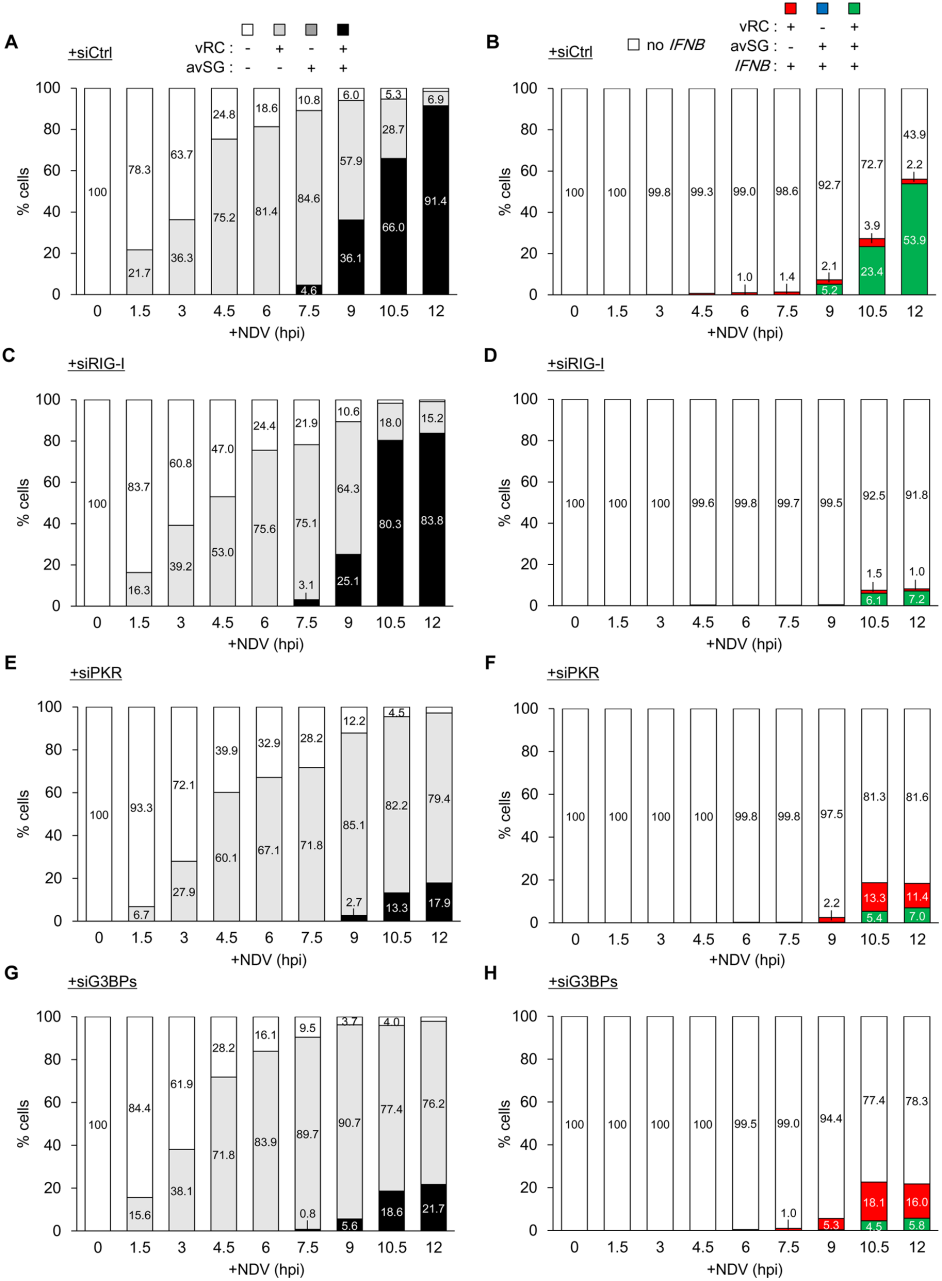
Quantitative analysis of vRC, avSG, and *IFNB* mRNA expression in HeLa cells knocked down for RIG-I, PKR, and G3BPs. **(A-H)** HeLa cells were transfected with siRNAs; siCtrl, siRIG-I, siPKR, or siG3BPs (siG3BP1 and siG3BP2). After transfection, the cells were infected with NDV (MOI = 1) for the indicated time up to 12 hours. Three hundred cells at each time point of infection were counted by MetaMorph software. Cells were categorized according to the existence of vRC, avSG, and *IFNB* mRNA, as shown on the top. Percentages were indicated inside the data bar.

**Fig 6:**
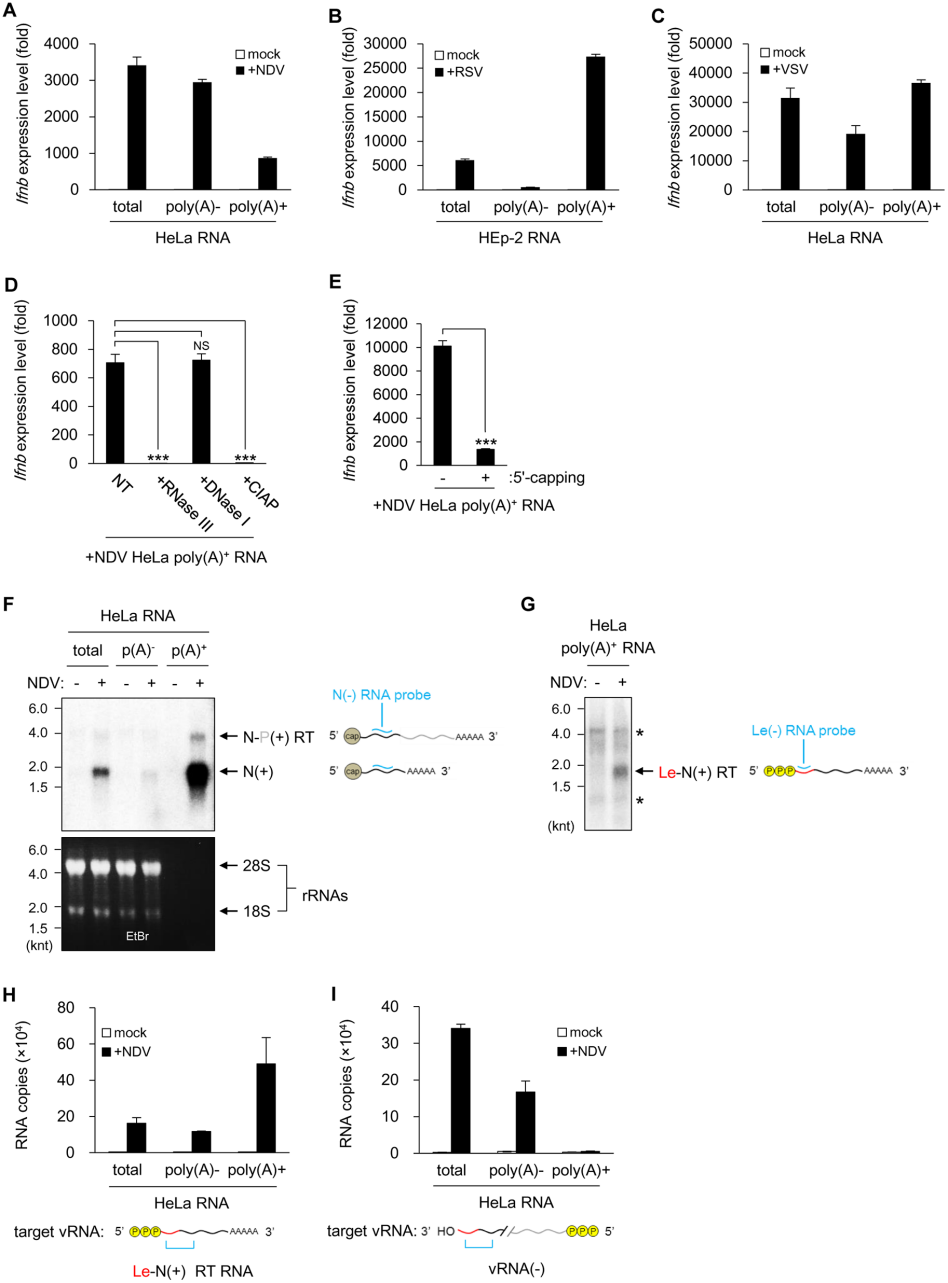
Uncapped, polyadenylated viral transcript induced *IFNB* gene expression. **(A-C)** Total RNA of mock treated or NDV-infected (12 hpi, MOI = 1) HeLa cells (A), RSV-infected (60 hpi, MOI = 1) HEp-2 cells (B), or VSV-infected (12 hpi, MOI = 1) HeLa cells (C) was separated into poly(A)^-^ and poly(A)^+^ RNA fractions by oligo(dT)-combined latex beads and transfected into MEFs (1×10^5^ cells were transfected with 200 ng RNA). *Ifnb* mRNA expression levels were measured by RT-qPCR. Data are represented as means ±SD. **(D and E)** The Poly(A)^+^ RNA fraction from NDV-infected (12 hpi, MOI = 1) HeLa cells was mock treated (NT) or treated with RNase III, DNase I, CIAP (D), or 5’-capping enzyme of Vaccinia virus (E) and then transfected to MEFs (1×10^5^ cells were transfected with 200 ng RNA). *Ifnb* mRNA expression levels were measured by RT-qPCR. Data are represented as means ±SD (t-test: **p<0.05, NS = not significant). **(F)** NDV vRNA(+) detection by strand-specific northern blotting. Total, poly(A)^-^, and poly(A)^+^ RNA (each 0250 ng) from mock treated or NDV-infected (12 hpi, MOI = 1) HeLa cells were separated on a denaturing agarose gel. An ethidium bromide (EtBr)-stained gel was shown in the bottom. vRNA(+) was detected by blotting with an N-specific RNA probe (N(-)). Positions of N vmRNA (N(+)) and N-P read-through RNA (N-P(+) RT) are shown. knt = kilo nucleotide. RNA probe and target vRNA(+) are illustrated alongside. **(G)** Poly(A)^+^ RNA from mock treated or NDV-infected (12 hpi, MOI = 1) HeLa cells were subjected to strand-specific northern blotting using a leader-specific RNA probe (Le(-)). The position of Le-N read-through RNA is shown (Le-N(+) RT). *non-specific band. RNA probe and target vRNA(+) are illustrated alongside. **(H and I)** Total, poly(A)^-^, and poly(A)^+^ RNA (each 200 ng) from mock treated or NDV-infected (12 hpi, MOI = 1) HeLa cells were subjected to strand-specific RT-qPCR (ssRT-qPCR) targeting Le-N(+) read-through RNA (H) or targeting vRNA(-) (I). Primers used are listed in Table in S1 Table. Data are represented as means ±SD. Target vRNAs in the assay are illustrated below.

We blocked the formation of SG in order to elucidate the significance of avSG in *IFNB* gene expression. We knocked down the expression of PKR, which has been shown to act as a sensor for viral infection in order to trigger the formation of avSG [20]. We also targeted G3BP proteins (G3BP1 and G3BP2: G3BPs), which are known to be critical for the assembly and maintenance of SG [17,34,35]. We knocked down RIG-I for comparison. A western blotting analysis confirmed the efficient knockdown of the targets (Fig 4A). The knockdown did not interfere with IFNAR-mediated STAT1 phosphorylation (S9 Fig). These cells were infected with NDV and *IFNB* gene expression was examined by RT-qPCR (Fig 4B). As expected, the knockdown of RIG-I impaired *IFNB* gene expression and the treatment with siPKR or siG3BPs also inhibited *IFNB* gene induction. The kinetics of vRC, avSG, and *IFNB* mRNA induction were subsequently examined in these cells (Fig 4C). These results were quantified as in Fig 2 (Fig 5). In control cells at 12 hpi, vRC were observed in almost all cells (light gray + black, 97–99%). Knockdown of RIG-I, PKR or G3BPs did not decrease the number of vRC-positive cells (light gray + black, Fig 5C, 5E and 5G). The knockdown of RIG-I markedly blocked *IFNB* mRNA (Fig 5D, red + green) expression (56.1 to 8.2% at 12 hpi); however, the number of avSG-positive cells (Fig 5C, black) remained constant (91.4 to 83.8%). The knockdown of PKR strongly inhibited the formation of avSG (Fig 5E, black) (91.4 to 17.9%) and decreased the number of *IFNB* mRNA-positive cells (Fig 5F, red + green) (56.1 to 18.4%). The knockdown of G3BPs also inhibited avSG (Fig 5G, black) (91.4 to 21.7%) and decreased the number of *IFNB* mRNA-positive cells (Fig 5H, red + green) (56.1 to 21.8%). Concomitant with the inhibition of avSG, the number of cells exhibiting vRC and *IFNB* mRNA increased, suggesting that these cells failed to develop into triple positive, high *IFNB* gene-expressing cells (Figs 5F and 6H). We also examined the expression of *ISG20*, *ISG56*, and *CXCL10* genes by RT-qPCR at 12 hpi (S10 Fig). The expression of these genes was dependent on RIG-I, PKR, and G3BPs. These results strongly suggested that avSG contributed to the robust *IFNB* gene expression observed at approximately 12 hpi.

### Viral poly(A)^+^ RNA stimulated *IFNB* gene induction

We found that avSG contained vRNA(+), but not vRNA(-) (Fig 1C) or vdsRNA (S4 Fig). We suspected that vRNA(+) activated RIG-I in avSG, culminating in *IFNB* gene expression at 12 hpi. Since poly(A)^+^ vmRNA is major vRNA(+), we isolated poly(A)^+^ RNA from mock and NDV infected cells. These RNA fractions were tested for the induction of *Ifnb* mRNA in mouse embryonic fibroblasts (MEFs). RNA fractions extracted from uninfected cells did not activate the *Ifnb* gene (Fig 6A), whereas both poly(A)^+^ and poly(A)^-^ RNA extracted from NDV-infected cells exhibited strong *Ifnb*-inducing activity. In addition to *Ifnb*, *Isg56* and *Cxcl10* genes were also activated by these RNA fractions (S11A and S11B Fig). It was unexpected that the poly(A)^+^ RNA fraction, corresponding to the mRNA fraction, exhibited stimulatory activity. We further tested other viruses belonging to *Mononegavirales*, RSV (Fig 6B) and VSV (Fig 6C). The results revealed that poly(A)^+^ RNA from RSV and VSV also exhibit strong stimulatory activity, particularly, majority of the stimulatory activity resides in poly(A)^+^ RNA in RSV-infected cells. This activity was sensitive to RNase III or CIAP, but not DNase I, suggesting that this activity resides in poly(A)^+^ RNA species with a secondary structure and end phosphate moieties, such as 5’-triphosphate (Fig 6D and S11C and S11D Fig). To examine the presence of 5'-triphosphate in the stimulatory RNA, the RNA fraction was subjected to reaction with capping enzyme of Vaccinia virus, which adds cap to 5'-triphosphate end. Capping reaction diminished stimulatory activity of the RNA (Fig 6E), therefore we speculate that the stimulatory activity is from remaining 5'-triphosphate -containing RNA.

### The NDV Le-N read-through transcript retains IFN-β-inducing activity

As described in the Introduction, transcription of NDV occurs in the order of 3’-Le-N-P-M-F-HN-L-Tr-5’. The first transcript Le possesses 5'-triphosphate and is devoid of 3’-poly(A). However, transcripts for N, P, M, F, HN and L possess 5'-cap and 3’-poly(A). We analyzed viral N mRNA by strand-specific northern blotting (Fig 6F). RNA extracted from NDV-infected HeLa cells was fractionated into poly(A)^+^ and poly(A)^-^ fractions. Ethidium bromide staining revealed the virtual absence of ribosomal RNA in poly(A)^+^ RNA fraction, demonstrating successful fractionation (Fig 6F, bottom). As expected, N mRNA (expected size 1.7 kilonucleotides, knt) is enriched in poly(A)^+^ RNA fraction (Fig 6F, top). This probe detected additional slow migrating RNA of 3.8 knt. We suspected that this larger RNA was read-through N mRNA, an extended transcript covering the N and P genes [36]. Many types of viruses belonging to *Mononegavirales* have been shown to produce read-through transcripts [37–39]. The result of Fig 6F inspired us to examine the presence of Le-N read-through transcript, because such an RNA possesses both 5'-triphosphate and 3'-poly(A) [38]. To detect Le-N read-through RNA, we performed strand-specific northern analysis using Le-specific RNA probe (Fig 6G). Poly(A)^+^ RNA from NDV-infected cells exhibited 1.7 knt signal by this probe. Le RNA (55 nt) was undetectable in this gel due to its small size.

**Fig 7:**
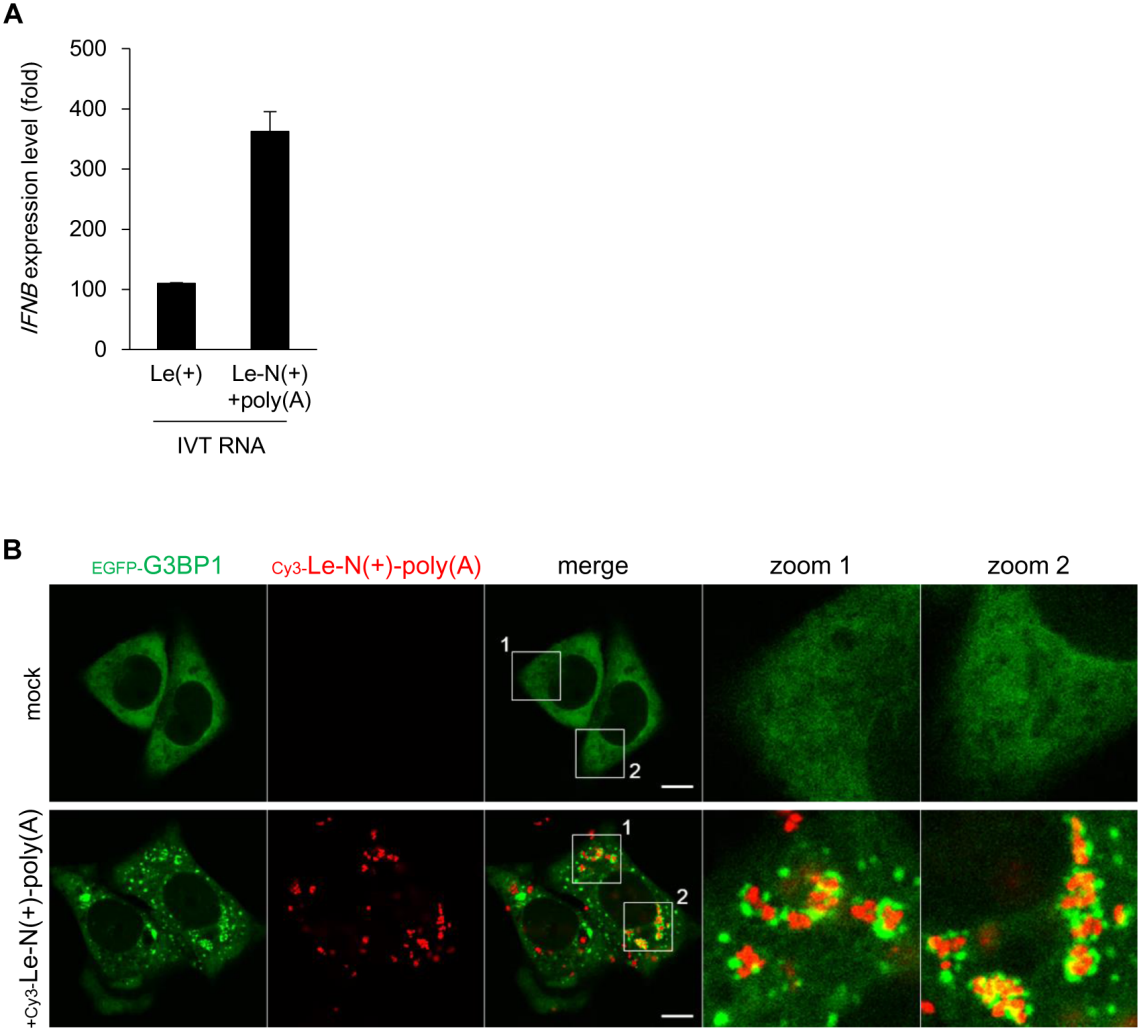
*In vitro* transcribed Le-N fusion RNA induced *IFNB* gene expression and SG formation. **(A)** RNA corresponding to NDV Le RNA (Le(+)) and Le-N read-through RNA (Le-N(+)+poly(A)) were synthesized *in vitro*. These RNA preparations (200 ng) were tested for *IFNB* gene expression by transfecting to FLAG-RIG-I/HeLa cells (2×10^5^ cells). *IFNB* mRNA expression levels were measured by RT-qPCR. Data are represented as means ±SD. **(B)** Cy3-labeled Le-N(+)+poly(A) (40 ng) was synthesized *in vitro* and transfected into EGFP-G3BP1/HeLa cells (0.25×10^5^ cells). After 6 hours, cells were treated with 10 μM chloroquine for 1 hour to enhance transfection efficiency. Cells were fixed and visualized for EGFP-G3BP1 (green) and Cy3-labelled RNA (red). Enlarged images of boxed areas are shown (zoom 1 and 2).

To quantify Le-N read-through RNA, we used a protocol for strand-specific RT-qPCR [40]. This method provides specific detection of the target RNA (S12 Fig). First, we quantified positive strand Le-N sequence (Fig 6H). As expected, Le-N was enriched in poly(A)^+^ fraction. Next, we quantified negative strand Le-N sequence to exclude a possibility that the stimulatory activity is from vmRNA partially hybridized with negative-sense strand (Fig 6I). The result revealed that negative strand Le-N sequence was hardly detectable in poly(A)^+^ fraction, excluding the possibility mentioned above. Interestingly, this technique allowed us to detect similar read-through products (Le-NS1, RSV; Le-N, VSV) in cells infected with RSV and VSV (S13 Fig). Finally, we synthesized RNA corresponding to Le and the Le-N read-through RNA and examined their IFN-inducing activity (Fig 7A). *In vitro* product of Le-N read-through RNA with 3’-poly(A)^+^ as well as Le RNA were capable of inducing *IFNB* gene activation. We monitored Cy3-labeled Le-N read-through RNA after transfection. The transfection induced formation of SG containing G3BP1 and Cy3 signal partially co-localized with the SG (Fig 7B). Here, Cy3 signal did not completely coincided with SG, presumably because most Cy3-RNA resided within endosomes and the portion of the RNA escaped into cytoplasm induced SG. The results suggest that Le-N read-through RNA is capable of inducing avSG and the following induction of *IFNB* gene.

## Discussion

The present study revealed that NDV-derived vRNA species were sensed by two distinct mechanisms. NDV infection resulted in the generation of vRC as early as 1.5 hpi and their number subsequently increased (Fig 2B). At 6 hpi, *IFNB* mRNA was detected in cells exhibiting vRC. vRC contained vRNAs (-, +), vdsRNA, and RIG-I (Figs 1B and 3A and S4 Fig). These results suggested that vRC are a locale in which vRNA is sensed by RIG-I and triggers IFN-inducing signal in the early stages of the infection (before 7.5 hpi). At 7.5 hpi, avSG were detected with concomitant increases in *IFNB* mRNA levels (Fig 2C). Since RIG-I also co-localized with avSG (Fig 3A) and avSG were strongly correlated with the culmination of *IFNB* gene induction at >9 hpi, these results suggested that avSG were a second locale for vRNA detection by RIG-I. This is consistent with the result that most (77 to 92%) cells expressing *IFNB* mRNA were positive for vRC and avSG after 7.5 hpi (Fig 2C) and that the inhibition of avSG markedly attenuated *IFNB* gene expression (Figs 4B and 5). Immunostaining results clearly indicated that vRC and avSG made contact with IPS-1 (Fig 3C and S8 Fig), suggesting signal transduction to IPS-1 from two distinct types of granules.

The transfection of RNA extracted from control and NDV-infected cells showed that NDV-derived vRNA activated the *IFNB* gene by transfection (Fig 6A). We detected vRNAs including vdsRNA in vRC (Fig 1B and S4 Fig), consistent with its function for vgRNA synthesis by viral replicase. However NDV-induced avSG selectively contained vRNA(+), and, hence, vmRNA or viral complementary RNA (vcRNA), a full-length template for the viral genome. This is in contrast to influenza A virus (IAV)-infected cells, in which vgRNA was shown to be localized within avSG [20]. avSG induced by infection with encephalomyocarditis virus were previously reported to contained vdsRNA [35]. These findings indicated that the mechanism underlying the induction of avSG and the vRNA content of avSG depended on virus types. We herein revealed that NDV, as well as other viruses belonging to *Mononegavirales* including RSV and VSV, produced Le-NS1/N fusion RNA with 3’-poly(A) (Fig 6G and 6H and S13C and S13F Fig). The Le transcript has been shown to contain 5’-triphosphate [23,41], and synthetic RNA corresponding to the Le-N strongly activated the *IFNB* gene (Fig 7A). On the other hand, we were unable to detect vcRNA, presumably because of its low abundance in addition to the low sensitivity of our detection. These results prompted us to speculate that viral Le-N RNA synthesized in vRC was transported to avSG and detected by RIG-I.

Host mRNA generally conforms to a certain format; therefore, it is not sensed as non-self. Since mRNA with m^7^G-cap is not sensed by RIG-I, the acquisition of a cap is a major strategy of viruses to escape immune detection. The importance of additional methylation adjacent to the cap has recently been reported [42,43]. In the present study, we demonstrated that RIG-I specifically recognized the uncapped vRNA of NDV generated by Le-N read-through transcription. The generation of uncapped vRNA through transcriptional read-through is not limited to NDV, it is commonly found in other negative-strand RNA viruses such as Sendai virus, measles virus (MeV), and vesicular stomatitis virus [37–39], suggesting that sensing such uncapped vRNA is an important strategy of antiviral innate immunity. In the case of MeV, 5’-triphosphate-ended Le RNA is considered to be a signature of activating RIG-I for IFN induction, and the association of RIG-I with the 5’ end of transcript covers the Le to N regions, corresponding to the Le-N read-through transcript [41,44]. In future studies, it will be interesting to explore the general impact of an abortive Le-N read-through transcript, which is exclusively produced by viruses belonging to *Mononegavirales*, on the RIG-I-driven IFN pathway.

Our results suggested that RIG-I detected vRNA within vRC in the early stages of infection and triggered the induction of IFN. However, this early response was more limited than the late response involving avSG, thereby suggesting the more efficient sensing of vRNA in avSG. PKR is known to be essential for the formation of avSG induced by NDV (Fig 4) and other viruses [18]. In MeV infection, PKR was shown to correlate positively with both avSG formation and IFN expression [45–47]. DHX36, another DExD/H-box helicase, has been shown to cooperate with PKR for the formation of avSG and subsequent viral RNA sensing by RIG-I [21]. Pumilio proteins (PUM1 and 2) are known to cooperate with LGP2 to sense vRNA in order to trigger IFN-inducing signaling [48]. PKR, DHX36, and PUM1 and 2 are specifically recruited to avSG. A critical signaling molecule for IFN induction, TRIM25, was shown to be specifically recruited to avSG [21]. Taken together, these findings and the results of the present study demonstrated that avSG serves as a platform for the efficient sensing of vRNA through the recruitment of critical signaling molecules.

In summary, the virus sensing in the cytoplasm involves more than a simple interaction between sensor molecules and vRNAs; a more complex mechanism including various RNA binding proteins and stress response machinery is responsible for detecting various vRNA structures generated by the replication of different viruses.

## Materials and Methods

### Cells

HeLa (#CCL-2.2, ATCC), FLAG-RIG-I/HeLa (derived from HeLa; #CCL-2.2, ATCC), FLAG-IPS-1/HeLa (derived from HeLa; #CCL-2.2, ATCC) [33], EGFP-G3BP1/HeLa (derived from HeLa; #CCL-2.2, ATCC) [35], HEp-2 (#CCL-23, ATCC), BHK21 (#CCL-10, ATCC) cells, and MEFs (isolated from embryos under C57BL/6 background, Japan SLC, Inc.) were maintained in Dulbecco’s Modified Eagle’s Medium (DMEM) (Nacalai Tesque) supplemented with 10% Fetal Bovine Serum (FBS) (BioWest) and 1% Penicillin-Streptomycin Mixed Solution (100 U/ml and 100 μg/ml respectively) (Nacalai Tesque).

### Virus and infection

NDV (strain Miyadera/51) was inoculated into 9-day embryonated chicken eggs and incubated for 2 days at 37°C, followed by overnight incubation at 4°C. Allantoic fluid containing NDV was collected from dead eggs. RSV (strain Long, ATCC VR-26) and VSV (strain Indiana, M mutant) were propagated in HEp-2 cells and BHK21 cells respectively, and culture supernatant was collected. The virus titer was determined by a plaque assay using HEp-2 cells. Virus was added to cells at a multiplicity of infection (MOI) of 1. After 1-hour incubation, the medium was replaced with fresh DMEM and incubated for the indicated hours of infection.

### Arsenite, RNase, and MitoTracker treatments

Sodium arsenite, and ribonuclease (RNase) A were purchased from SIGMA-ALDRICH. ShortCut RNase III was purchased from New England Biolabs. MitoTracker Red CMXRos was purchased from Thermo Fisher Scientific. Cells were treated as described in the figure legends.

### Immunofluorescence

Cells were fixed with 4% paraformaldehyde solution for 10 minutes at room temperature. A 0.5% Triton X-100 solution was added to the cells for permeabilization and incubated for 5 minutes at room temperate. Regarding blocking, 0.5 mg/ml BSA solution in PBST (PBS containing 0.04% Tween-20) was added to the cells and incubated for 30 minutes at room temperature. Primary antibodies were diluted in 0.5 mg/ml BSA/PBST and added to the cells, then incubated overnight at 4°C. After washing with PBST, secondary antibodies were added to the cells at a 1:1,000 dilution in 0.5 mg/ml BSA/PBST, and incubated for 1 hour at room temperature. After washing with PBST, 1 μg/ml DAPI solution in PBS was added to the cells to stain the nucleus. Cells were briefly rinsed with PBS, and then mounted with Fluromount-G (SouthernBiotech). Images were taken by the confocal laser scanning microscope, TCS-SP8 (Leica Microsystems). The primary antibodies used were; anti-NDV-N mouse mAb (provided by Dr. T. Sakaguchi, Hiroshima University in Japan), anti-TIA-1 goat pAb (#sc-1751, Santa Cruz Biotechnology), anti-TIAR rabbit mAb (#8509, Cell Signaling Technology), anti-TIAR goat pAb (#sc-1749, Santa Cruz Biotechnology), anti-G3BP1 mouse mAb (#sc-365338, Santa Cruz Biotechnology), anti-eIF3η (#sc-16377, Santa Cruz Biotechnology), anti-FLAG M2 mouse mAb (#F1804, SIGMA-ALDRICH), and anti-dsRNA/J2 mouse mAb (English & Scientific Consulting Kft.). Anti-IPS-1 guinea pig pAb was provided by Dr. I. Julkunen. Anti-RIG-I and anti-NDV-L antibodies were originally generated by immunizing rabbits with synthetic peptides corresponding to amino acids 793–807 of human RIG-I and 1160–1183 of NDV-L, respectively. The secondary antibodies used were; Alexa Fluor 488 donkey anti-rabbit IgG H+L (#A-21206), Alexa Fluor 488 donkey anti-mouse IgG H+L (#A-21202), Alexa Fluor 488 Donkey anti-Goat IgG H+L (#A-11055), Alexa Fluor 594 Donkey anti-Rabbit IgG H+L (#A-21207), Alexa Fluor 594 Donkey anti-Mouse IgG H+L (#A-21203), Alexa Fluor 633 Goat anti-Mouse IgG H+L (#A-21050), Alexa Fluor 633 Donkey anti-Goat IgG H+L (#A-21082), and Alexa Fluor 647 Donkey anti-Goat IgG H+L (#A-21447), all purchased from Life Technologies.

### RNA-FISH

RNA-FISH assay was performed using the QuantiGene ViewRNA ISH Cell Assay Kit (Affymetrix) according to manufacturer’s instructions as below. Cells were fixed in 4% paraformaldehyde solution for 30 minutes and permeabilized for 5 minutes with detergent solution. Protease solution was added to the cells at a 1:4,000 dilution in PBS and incubated for 10 minutes. After washing with PBS, the cells were incubated with a probe set at a 1:25 dilution for 3 hours at 40°C. The cells were further and independently incubated with a pre-amplifier, amplifier, and label probe (all at a 1:25 dilution) for 30 minutes at 40°C. After washing with PBS, the cells were subjected to an immunofluorescence assay. The probe sets used were; *NDV-F*(-) (#VF1-15407), *NDV-N*(+) (#VF4-15408), and human *IFNB1* (#VA1-11281), all purchased from Affymetrix.

### Computer-based statistical cell analysis

Confocal micrographs of NDV-infected or arsenite-treated cells were subjected to the automatic analysis module (Multi Wavelength Cell Scoring) of MetaMorph Software v7.7 (Molecular Devices) in order to count vRC and SG speckles and *IFNB* mRNA dots. Briefly, the total cell number was first determined by counting the number of nuclei in the DAPI channel. A single cell area was segmented from the TIAR or eIF3η channel in reference to the intercellular boundary of the cytoplasmic staining area. The numbers of vRC (N), SG (TIAR or eIF3η), and *IFNB* mRNA were counted from each channel.

### RNAi gene knockdown

siRNAs for *RIG-I/DDX58* (HSS119008), *PKR* (HSS108571), *G3BP1* (HSS115444), *G3BP2* (HSS114988), and a negative control (#12935–300), purchased form Life Technologies, were transfected into 1×10^5^ HeLa cells at a final concentration of 10 nM using Lipofectamine RNAiMAX Reagents (Life technologies). 24 hours after transfection, the cells were transferred to new culture plates with fresh DMEM. After being incubated for a further 24 hours, cells were subjected to the following experiments.

### Western blotting

Cells were lysed with ice-cold NP-40 lysis buffer (50 mM Tris-HCl [pH 8.0], 150 mM NaCl, 1% NP-40, 1 mM sodium orthovanadate, 1 mM PMSF, and 0.1 mg/ml leupeptin). After centrifugation, the supernatant was collected, mixed with an equal volume of 2× SDS sample buffer (125 mM Tris-HCl [pH 6.8], 4% SDS, 20% glycerol, 0.01% BPB, and 10% 2-mercaptoethanol), and boiled for 5 minutes. The sample corresponding to a protein amount of 30 μg was applied to 5–20% gradient e-PAGEL (ATTO), separated by a standard SDS-PAGE method, and then transferred onto an Immobilon-P PVDF membrane (MILLIPORE). The membrane was incubated in Tris-buffered saline with 0.1% Tween-20 (TBST) containing 5% skimmed milk for 30 minutes at room temperature for blocking. The membrane was incubated with a primary antibody diluted in the blocking buffer overnight at 4°C. After washing with TBST, the membrane was incubated with an AP-conjugated secondary antibody diluted in the blocking buffer for 1 hour at room temperature. After washing with TBST, protein bands were visualized using the BCIP-NBT Solution Kit for Alkaline Phosphate Stain (Nacalai Tesque) or ECL Prime Western Blotting Detection Reagent (GE Healthcare). The primary antibodies used were; anti-PKR mouse mAb (#sc-6282, Santa Cruz Biotechnology), anti-phospho-PKR rabbit pAb (#ab13447, Abcam), anti-G3BP2 goat pAb (#sc-161612), anti-STAT1 rabbit pAb (#9172, Cell Signaling Technology), anti-phospho-STAT1rabbit pAb (#9172, Cell Signaling Technology), and anti-β-Actin mouse mAb (#A2228, SIGMA ALDRICH). The secondary antibodies used were; goat anti-rabbit IgG-AP (#sc-2007, Santa Cruz Biotechnology), goat anti-mouse IgG-AP (#sc-2008, Santa Cruz Biotechnology), anti-rabbit IgG, HRP-linked (#7074, Cell Signaling Technology), and anti-mouse IgG, HRP-linked (#7076, Cell Signaling Technology).

### Reverse transcription and quantitative PCR (RT-qPCR)

Total RNA was isolated from cells using TRIzol Reagent (Ambion), and treated with RNase-Free Recombinant DNase I (Roche Diagnostics). After phenol-chloroform extraction and ethanol precipitation, purified total RNA was subjected to cDNA synthesis using a High-Capacity cDNA Reverse Transcription Kit (Applied Biosystems). Gene expression levels were measured by the StepOnePlus Real-Time PCR system (Applied Biosystems) using the TaqMan Fast Universal PCR Master Mix (Applied Biosystems), and determined by the 2^-ΔΔCt^ relative quantitative method. The TaqMan probes used for measurements were; *IFNB1* (#Hs01077958_s1), *ISG20* (#Hs00158122_m1), *ISG56*/*IFIT1* (#Hs01911452_s1), *CXCL10* (#Hs01124251_g1), *Ifnb1* (#Mm00439552_s1), *Isg56*/*Ifit1* (#Mm00515153_m1), *Cxcl10* (#Mm00445235_m1), and eukaryotic *18S rRNA* (#4333760F), all purchased from Applied Biosystems. The probe for *NDV-N* was designed as below: 5'-GTCCGTATTTGACGAATACGAG-3' (forward primer), 5'-CAAGGGCAACATGGTTCCTC-3' (reverse primer), and 5'-TCAGGCAAGGTGCTC-3' (probe).

### RNA preparation and transfection

Poly(A)^+^ mRNA was isolated from the total RNA of mock/NDV-infected (12 hpi, MOI = 1) HeLa cells using the Oligotex-dT30 <Super> mRNA Purification Kit (TaKaRa) according to manufacturer’s instructions. Purification was repeated twice to yield a pure poly(A)^+^ mRNA fraction. The supernatant after centrifugation was subjected to ethanol precipitation in order to obtain a concentrated poly(A)^-^ RNA fraction. NDV gRNA was isolated from virus particles propagated in the embryonated chicken eggs, as described above. Allantoic fluid was centrifuged overnight at 15,000 rpm at 4°C, and the pellet was lysed using TRIZOL Reagent (Ambion) followed by isopropanol precipitation. 5’-triphosphate RNA was synthesized *in vitro* as reported previously [49]. RNA samples were treated with 1 U of ShortCut RNase III (New England Biolabs), 1 U of RNase-free DNase I recombinant (Roche), and 15 U of Calf Intestine Alkaline Phosphatase (Takara) at 37°C for 30 minutes, or 10 U of Vaccinia Capping Enzyme (New England Biolabs) according to the manufacturer’s instruction. After this treatment, RNA samples were purified by phenol-chloroform extraction and ethanol precipitation. Regarding RNA transfection, 200 ng of each RNA sample was transfected into 1×10^5^ MEFs or 2×10^5^ HeLa cells using Lipofectamine 2000 (Invitrogen) according to the manufacturer’s instruction.

### *In vitro* transcription

NDV gRNA isolated from virus particles by TRIZOL Reagent was subjected to reverse transcription, as described above. Synthesized cDNA was further subjected to PCR using primer sets including the T7 RNA polymerase promoter sequence (Table in S1 Table). PCR products were *in vitro* transcribed by the T7 RiboMAX Express Large Scale RNA Production System (Promega) according to manufacturer’s instructions. Ribo m^7^G cap analog (Promega) and Cy3-UTP (GE Healthcare) were included in the reaction for 5’-m^7^G capping and Cy3 labeling respectively. Poly(A) Tailing Kit (Ambion) was used for 3’-poly(A) modifications. Unincorporated nucleotides within the samples were removed by NucAway Spin Columns (Ambion). *In vitro* transcribed RNA samples were transfected, as described above.

### Northern blotting

A denaturing agarose gel was prepared at a final concentration of 1% (w/v) Agarose ME (Nacalai Tesque), 1× MESA (Dojindo), 2% formaldehyde (Nacalai Tesque), and 0.5 μg/ml ethidium bromide. A total of 250 ng of each RNA sample was mixed with an equal volume of Gel Loading Buffer II (Ambion) and incubated at 65°C for 15 minutes, followed by quick cooling on ice, and then electrophoresed in 1× MESA. The gel was transferred onto a nylon membrane Hybond-N (GE Healthcare) by a capillary blotting method using 10× SSC Buffer (Nacalai Tesque). After UV cross-linking, the membrane was pre-incubated in PerfectHyb Hybridization Solution (TOYOBO) at 65°C for 20 minutes. An RNA probe was added to the solution and incubated at 65°C overnight. The membrane was washed with 2× SSC (+0.1% SDS) and 0.2× SSC (+0.1% SDS) at 65°C for 15 minutes, and then irradiated onto Storage Phosphor Screen BAS-IP (GE Healthcare). Images were scanned using BAS-5000 Image Analyzer (Fujifilm). In order to prepare RNA probes (N(-) and Le(-)), NDV cDNA was subjected to PCR using primer sets including the T7 RNA polymerase promoter sequence (Table in S1 Table). PCR products were *in vitro* transcribed into [α-^32^P]-CTP-radiolabeled RNA probes by Riboprobe System-T7 (Promega). Unincorporated nucleotides within the samples were removed by NucAway Spin Columns (Ambion).

### Strand-specific RT-qPCR

Strand-specific RT-qPCR targeting vRNA was performed as introduced elsewhere [40]. In the RT step, cDNA complementary to the target vRNA was synthesized with the primer including “5’-tag”, of which sequence is unrelated to NDV, RSV, and VSV (Tables in S2, S3 and S4 Tables). After the reaction, RT sample was treated with 10 U of Exonuclease I (New England Biolabs) at 37°C for 1 hour to remove unincorporated primer, and then the reaction was inactivated at 60°C for 30 minutes. The tagged-cDNA was subjected to qPCR analysis with Fast SYBR Green Mater Mix (Thermo Fisher Scientific), using a specific primer set; primer corresponding to tag sequence and vRNA-specific primer (Tables in S1, S2 and S3 Tables). Standard curve was generated from ten-fold serial dilutions (10^10^, 10^9^, 10^8^, 10^7^, 10^6^, 10^5^, 10^4^, 10^3^ copies/μl) of vgRNA isolated from viral particles or *in vitro* synthesized vRNA.

## Supporting Information

S1 Fig Specificity of RNA-FISH. Mock-treated HeLa cells were fixed and subjected to FISH detection for NDV vRNA(+) (green) and vRNA(-) (red). Cells were also immunostained for N (white A) or TIAR (white B). Nuclei were co-stained with DAPI (blue). The white scale bar corresponds to 10 μm.
(TIF)

S2 Fig NDV infection induced formation of avSG. HeLa cells were either mock treated or infected with NDV (MOI = 1) for 6 and 12 hours and then immunostained for TIAR (green), G3BP1 (red), and eIF3η (white). Nuclei were stained with DAPI (blue). The boxed area of cell image at 12 hpi was enlarged and displayed on the upper left of the image. The white scale bar corresponds to 10 μm.
(TIF)

S3 Fig NDV infection induced two distinct granules vRC and avSG. HeLa cells were either mock treated or infected with NDV (MOI = 1) for 12 hours and immunostained for TIAR (green, A), L (green, B), G3BP1 (red), and TIA-1 (white). Nuclei were stained with DAPI (blue). The white scale bar corresponds to 10 μm.
(TIF)

S4 Fig NDV vdsRNA dominantly localized in vRC, but not SG. **(A and B)** HeLa cells were either mock treated or infected with NDV (MOI = 1) for 6 and 12 hours. (A) Cells were immunostained for L (green), vdsRNA (red), and TIAR (white). The boxed area was enlarged and displayed on the upper left of the image. (B) NDV vRNA(-) (red) and vRNA(+) (green) were detected by the RNA-FISH method. NDV vdsRNA was immunostained with a specific antibody (white). A merged image at the original magnification was shown in the rightmost panel. Nuclei were stained with DAPI (blue). The white scale bar corresponds to 10 μm.
(TIF)

S5 Fig Validation for specificity of an anti-dsRNA antibody. HeLa cells infected with NDV for 12 hours (MOI = 1) were fixed and permeabilized, and then treated with 200 μg/ml RNase A (at low NaCl concentration) or 30 units/ml RNase III. The cells were immunostained for L (green), vdsRNA (red), and TIAR (white). Nuclei were stained with DAPI (blue). The white scale bar corresponds to 10 μm.
(TIF)

S6 Fig Relationship between *IFNB* mRNA detected by RNA-FISH and nuclear IRF-3 and NF-κB. HeLa cells were either mock infected or infected with NDV (MOI = 1) for 12 hours. *IFNB* mRNA (green) was detected by the RNA-FISH method. IRF-3 (A) and p65/RelA (B) shown in red were immunostained with the respective antibodies. Nuclei were stained with DAPI (blue). The white scale bar corresponds to 10 μm.
(TIF)

S7 Fig FLAG-IPS-1 localized on mitochondria. FLAG-IPS-1/HeLa cells were either mock treated or infected with NDV (MOI = 1) for 12 hours. At 11.5 hpi, the medium was replaced with fresh medium containing 1 μM MitoTracker Red. The cells were immunostained for FLAG (green). Nuclei were stained with DAPI (blue). The white scale bar corresponds to 10 μm. The boxed area was enlarged and displayed on the right (zoom). As described by Onoguchi et al [33], NDV infection induced re-location of IPS-1, generating mitochondrion (red staining) partially devoid of IPS-1 (green).
(TIF)

S8 Fig Endogenous IPS-1 accumulated around both vRC and avSG. HeLa cells were either mock treated or infected with NDV (MOI = 1) for 12 hours. At 11.5 hpi, the medium was replaced with fresh medium containing 1 μM MitoTracker Red (A). The cells were then immunostained for IPS-1 (green, using a specific antibody produced in guinea pig), L (red), and TIAR (white). Nuclei were stained with DAPI (blue). The white scale bar corresponds to 10 μm. The boxed area was enlarged and displayed on the right (zoom). Partial co-localization between IPS-1 and L (vRC) or TIAR (avSG) is shown by displaying them in green and red, respectively (zoom).
(TIF)

S9 Fig IFNAR-mediated phosphorylation of STAT1 in siRNA transfected cells. HeLa cells were transfected with siRNAs; siCtrl, siRIG-I, siPKR, or siG3BPs (for G3BP1 and G3BP2) for 48 hours. The cells were then stimulated with IFN-β (1,000 U/ml) for the indicated time. Phosphorylation level of STAT1 was detected by western blotting.
(TIF)

S10 Fig Inhibition of avSG formation led to diminished expression of ISG. HeLa cells were transfected with siRNAs; siCtrl, siRIG-I, siPKR, or siG3BPs (siG3BP1 and siG3BP2). After transfection, the cells were mock treated or infected with NDV (MOI = 1) for 12 hours. Expression levels of *ISG20*, *ISG56*, and *CXCL10* mRNA were measured by qRT-PCR. Data are represented as means ±SD (t-test: ***p<0.01, **p<0.05, *p<0.1, NS = not significant).
(TIF)

S11 Fig Induction of ISG expression by NDV poly(A)^+^ RNA. **(A and B)** MEFs (2×10^5^ cells) were transfected with total, poly(A)^-^, or poly(A)^+^ RNA (200 ng) from mock/NDV-infected (MOI = 1) HeLa cells. *Isg56* and *Cxcl10* mRNA expression levels were measured by RT-qPCR. **(C and D)** Poly(A)^+^ RNA (200 ng) from NDV-infected (MOI = 1) HeLa cells was mock treated (NT) or treated with RNase III, DNase I, or CIAP, and then transfected to MEFs (2×10^5^ cells). *Isg56* and *Cxcl10* mRNA expression was quantified by RT-qPCR. Data are represented as means ±SD.
(TIF)

S12 Fig Validation of Strand-specific RT-qPCR for vRNAs. **(A-C)** 10^10^ copies of vgRNA isolated from the viral particles and *in vitro* synthesized RNA corresponding to vmRNA (N, NS1 and N for NDV, RSV and VSV, respectively) and read-through RNA (Le-N, Le-NS1 and Le-N for NDV, RSV and VSV, respectively) were subjected to strand-specific RT-qPCR (ssRT-qPCR) using specific primer sets (Tables in S2, S3 and S4 Tables). Percentage of the RNA copies of each target RNA was shown. Data are represented as means ±SD. The results showed specificity of ssRT-qPCR: probe for vRNA(-) only detected vgRNA; probe for Le-N/NS1(+) read-through RNA selectively detected Le-N/NS1 RNA but not N mRNA.
(TIF)

S13 Fig Strand-specific RT-qPCR analysis for RSV and VSV vRNAs. **(A-F)** Total, poly(A)^-^, and poly(A)^+^ RNA from mock treated, RSV-infected (60 hpi, MOI = 1) HEp-2 cells (A-C), or VSV-infected (12 hpi, MOI = 1) HeLa cells (D-F) were subjected to strand-specific RT-qPCR (ssRT-qPCR) targeting Le-NS1/N(-) as a portion of vgRNA, NS1/N(+) vmRNA, and Le-NS1/N(+) read-through RNA with specific primer sets (Tables in S3 and S4 Tables). Data are represented as means ±SD.
(TIF)

S1 Table Primers used for *in vitro* transcription. (TIF)

S2 Table Primer sets used for ssRT-qPCR targeting NDV vRNAs. (TIF)

S3 Table Primer sets used for ssRT-qPCR targeting RSV vRNAs. (TIF)

S4 Table Primer sets used for ssRT-qPCR targeting VSV vRNAs. (TIF)
