# Correction: Leader-Containing Uncapped Viral Transcript Activates RIG-I in Antiviral Stress Granules

**DOI:** 10.1371/journal.ppat.1005563

**Published:** 2016-04-05

**Authors:** Seong-Wook Oh, Koji Onomoto, Mai Wakimoto, Kazuhide Onoguchi, Fumiyoshi Ishidate, Takahiro Fujiwara, Mitsutoshi Yoneyama, Hiroki Kato, Takashi Fujita

The second affiliation for the first author is missing. Seong-Wook Oh is also affiliated with #3 Laboratory of Molecular and Cellular Immunology, Graduate School of Biostudies, Kyoto University, Kyoto, Japan

## References

[1] Oh S-W, Onomoto K, Wakimoto M, Onoguchi K, Ishidate F, Fujiwara T, et al (2016) Leader-Containing Uncapped Viral Transcript Activates RIG-I in Antiviral Stress Granules. PLoS Pathog 12(2): e1005444 doi: 10.1371/journal.ppat.1005444 26862753 10.1371/journal.ppat.1005444 PMC4749238

