# Supplementary figures and images for: Leader-Containing Uncapped Viral Transcript Activates RIG-I in Antiviral Stress Granules
Source: PLoS Pathog. 2016 Feb 10;12(2):e1005444. doi: 10.1371/journal.ppat.1005444 (PMC4749238; doi:10.1371/journal.ppat.1005444)

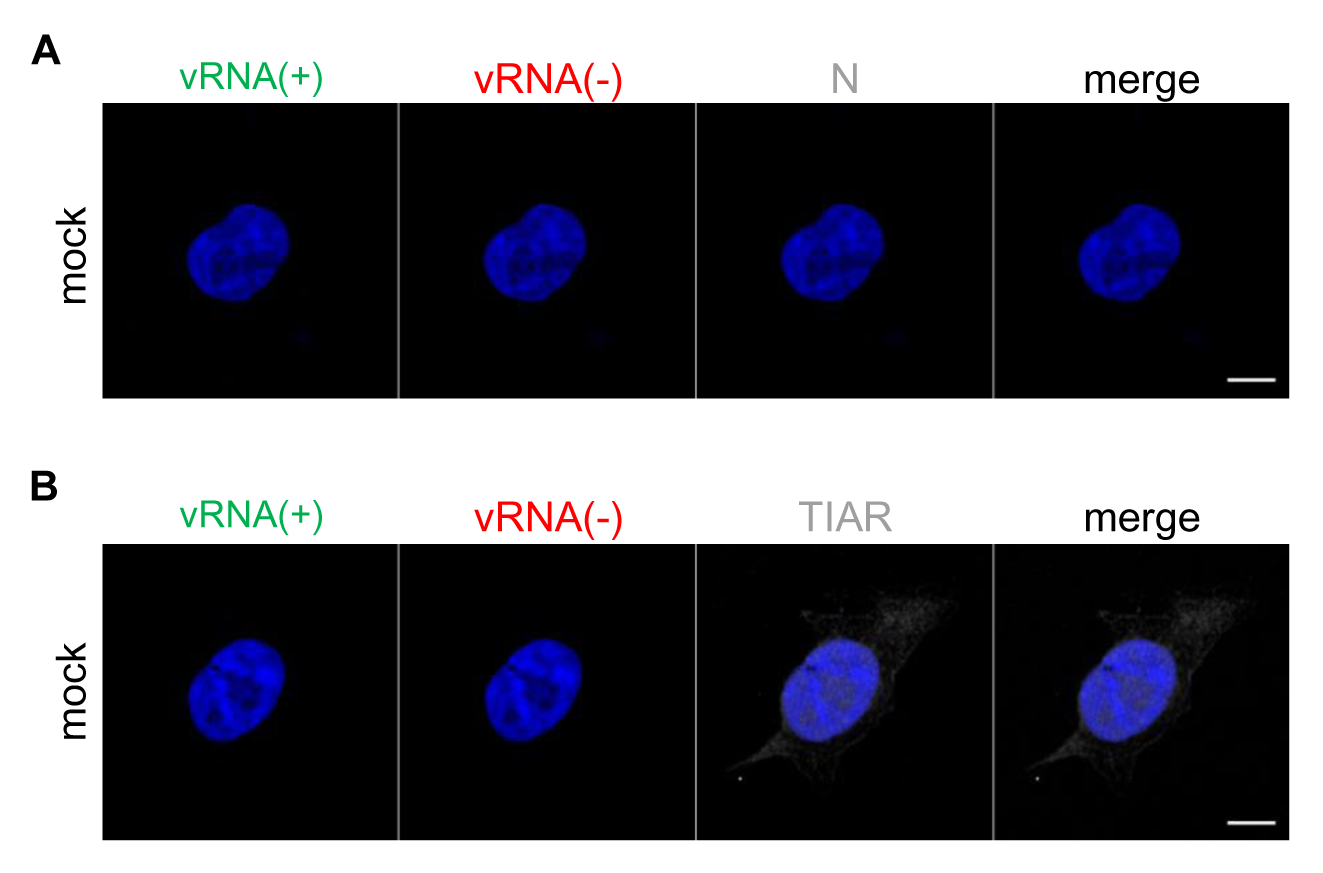

Supplement: S1 Fig — Mock-treated HeLa cells were fixed and subjected to FISH detection for NDV vRNA(+) (green) and vRNA(-) (red). Cells were also immunostained for N (white A) or TIAR (white B). Nuclei were co-stained with DAPI (blue). The white scale bar corresponds to 10 μm. (TIF) [file ppat.1005444.s001.tif]

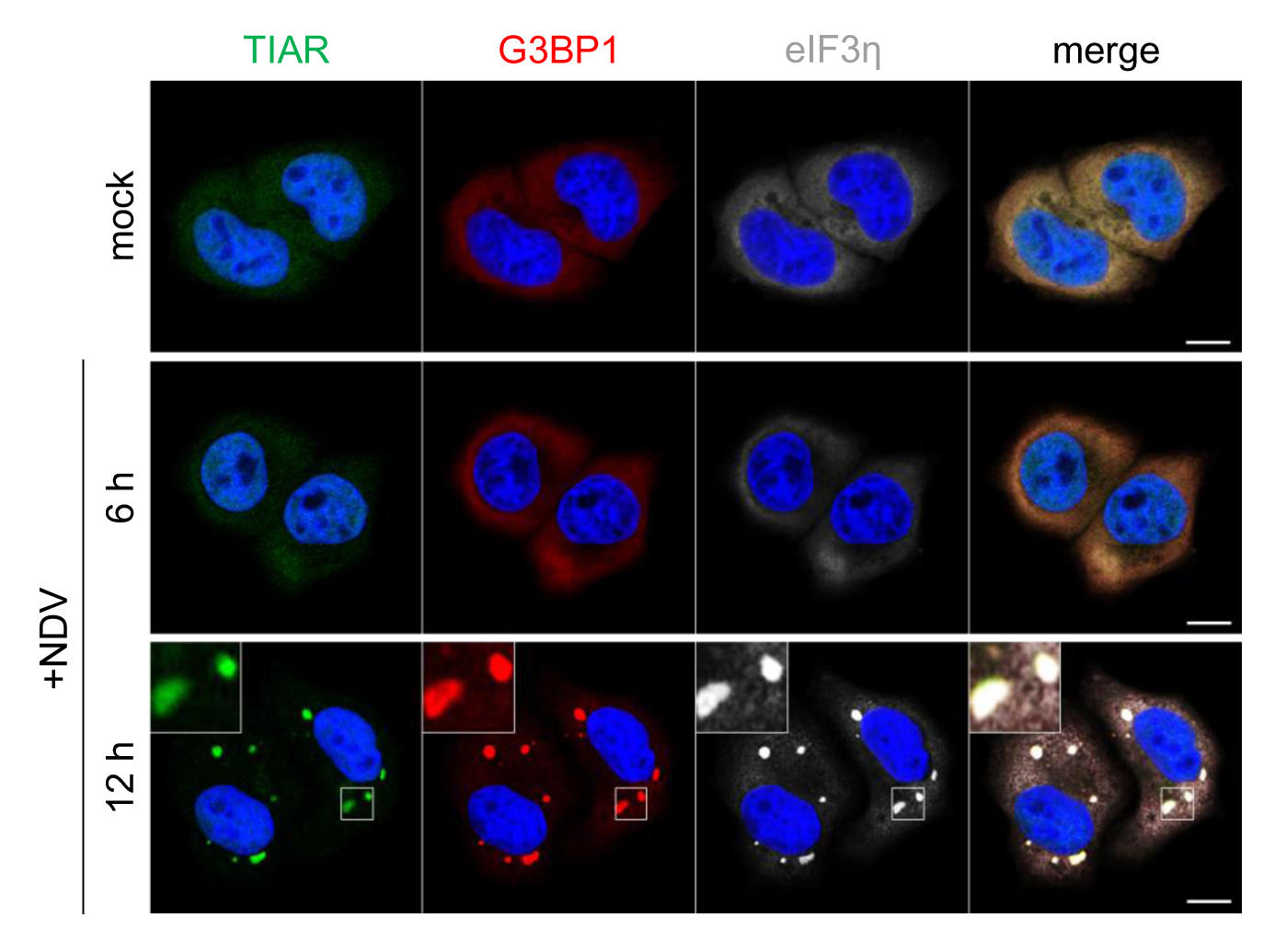

Supplement: S2 Fig — HeLa cells were either mock treated or infected with NDV (MOI = 1) for 6 and 12 hours and then immunostained for TIAR (green), G3BP1 (red), and eIF3η (white). Nuclei were stained with DAPI (blue). The boxed area of cell image at 12 hpi was enlarged and displayed on the upper left of the image. The white scale bar corresponds to 10 μm. (TIF) [file ppat.1005444.s002.tif]

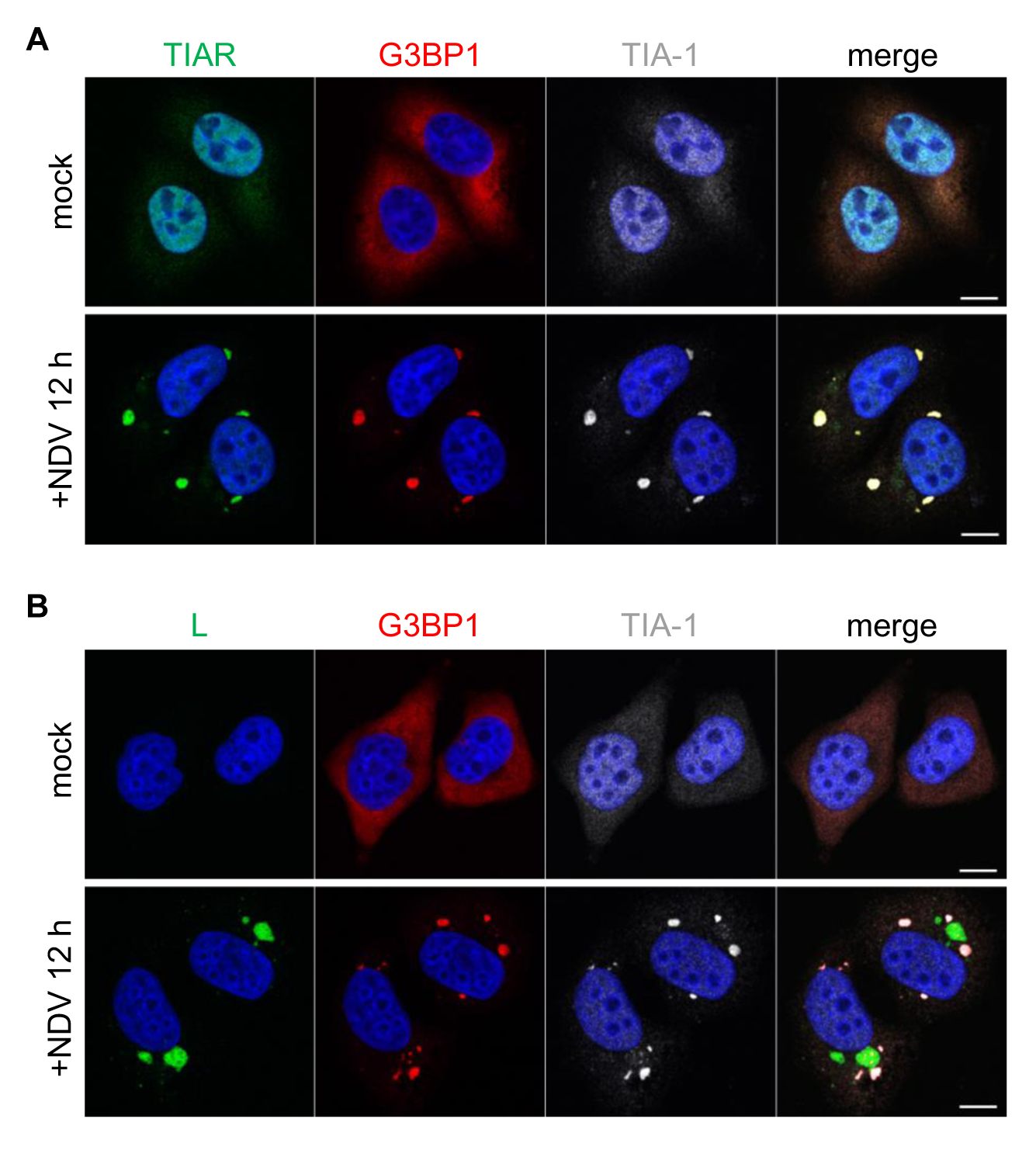

Supplement: S3 Fig — HeLa cells were either mock treated or infected with NDV (MOI = 1) for 12 hours and immunostained for TIAR (green, A), L (green, B), G3BP1 (red), and TIA-1 (white). Nuclei were stained with DAPI (blue). The white scale bar corresponds to 10 μm. (TIF) [file ppat.1005444.s003.tif]

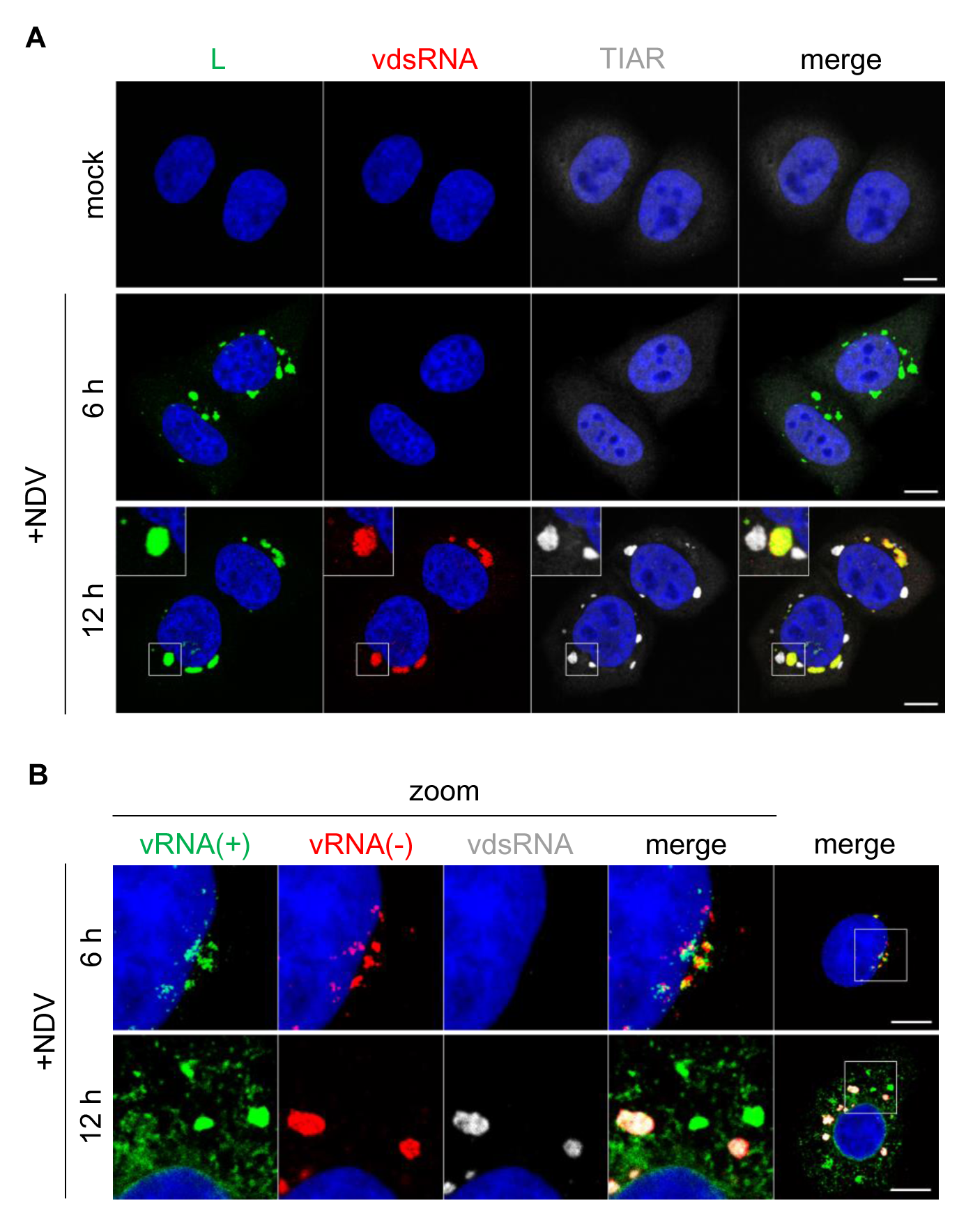

Supplement: S4 Fig — (A and B) HeLa cells were either mock treated or infected with NDV (MOI = 1) for 6 and 12 hours. (A) Cells were immunostained for L (green), vdsRNA (red), and TIAR (white). The boxed area was enlarged and displayed on the upper left of the image. (B) NDV vRNA(-) (red) and vRNA(+) (green) were detected by the RNA-FISH method. NDV vdsRNA was immunostained with a specific antibody (white). A merged image at the original magnification was shown in the rightmost panel. Nuclei were stained with DAPI (blue). The white scale bar corresponds to 10 μm. (TIF) [file ppat.1005444.s004.tif]

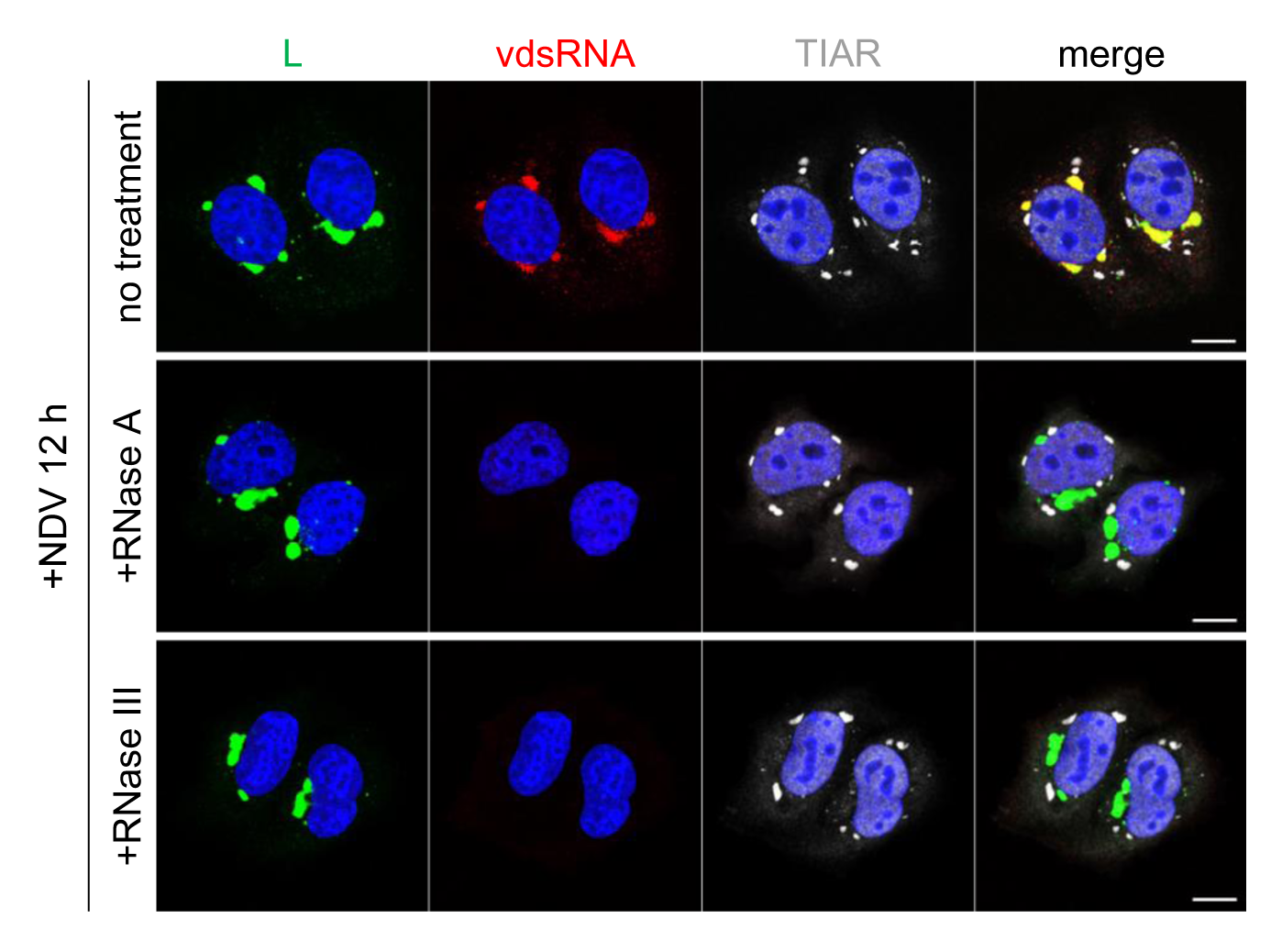

Supplement: S5 Fig — HeLa cells infected with NDV for 12 hours (MOI = 1) were fixed and permeabilized, and then treated with 200 μg/ml RNase A (at low NaCl concentration) or 30 units/ml RNase III. The cells were immunostained for L (green), vdsRNA (red), and TIAR (white). Nuclei were stained with DAPI (blue). The white scale bar corresponds to 10 μm. (TIF) [file ppat.1005444.s005.tif]

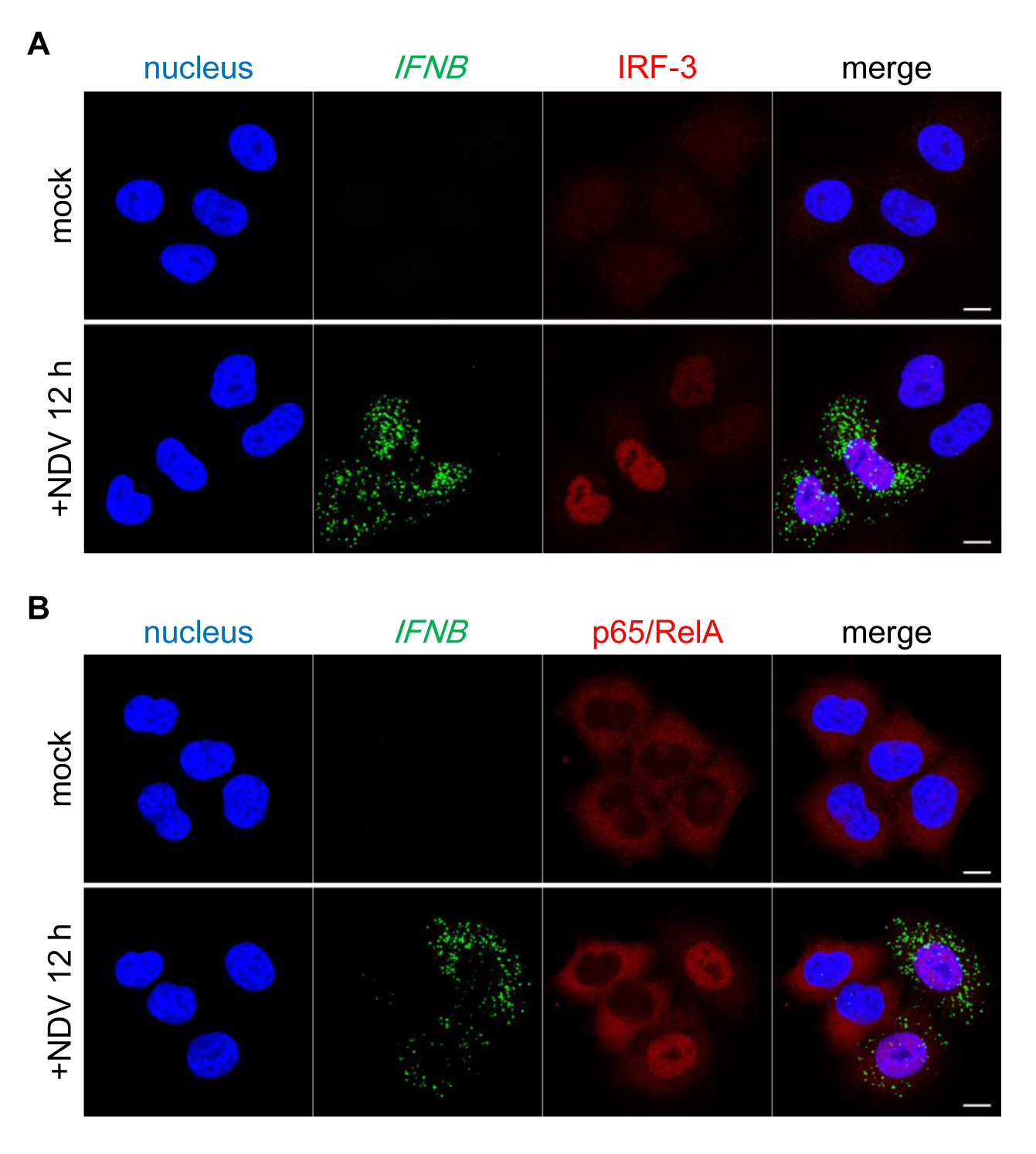

Supplement: S6 Fig — HeLa cells were either mock infected or infected with NDV (MOI = 1) for 12 hours. IFNB mRNA (green) was detected by the RNA-FISH method. IRF-3 (A) and p65/RelA (B) shown in red were immunostained with the respective antibodies. Nuclei were stained with DAPI (blue). The white scale bar corresponds to 10 μm. (TIF) [file ppat.1005444.s006.tif]

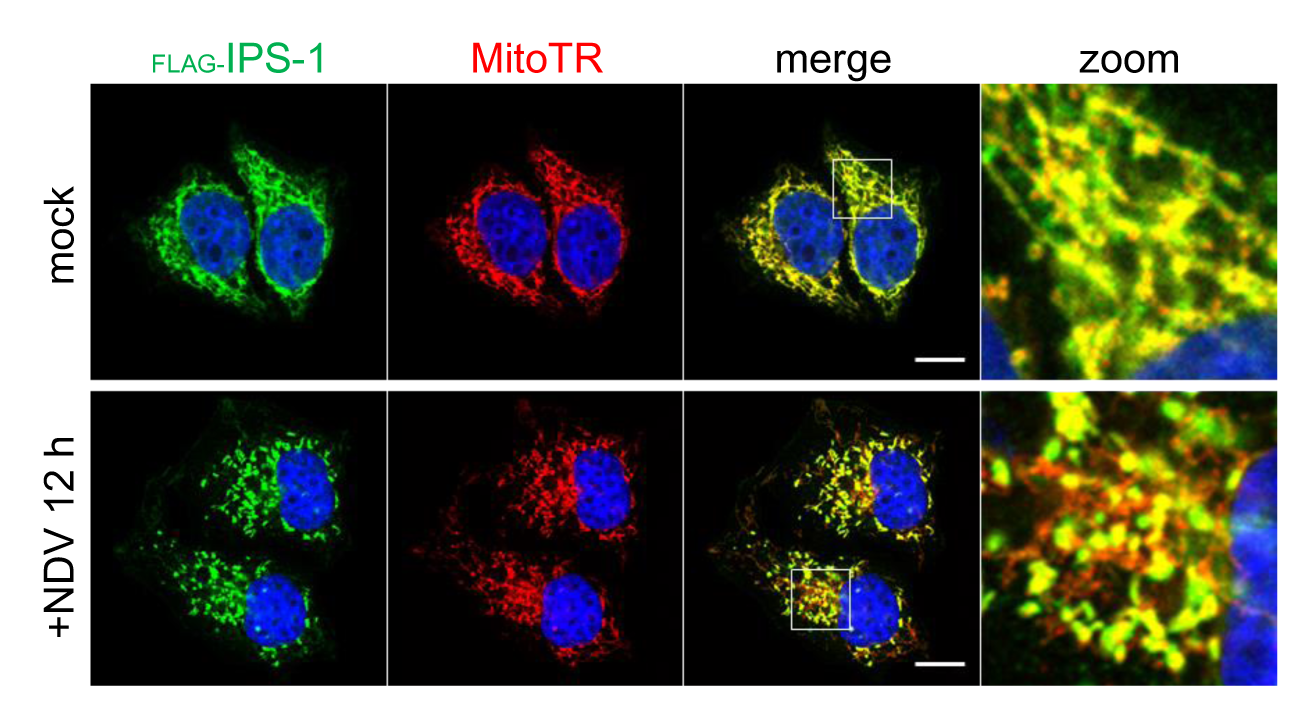

Supplement: S7 Fig — FLAG-IPS-1/HeLa cells were either mock treated or infected with NDV (MOI = 1) for 12 hours. At 11.5 hpi, the medium was replaced with fresh medium containing 1 μM MitoTracker Red. The cells were immunostained for FLAG (green). Nuclei were stained with DAPI (blue). The white scale bar corresponds to 10 μm. The boxed area was enlarged and displayed on the right (zoom). As described by Onoguchi et al [33], NDV infection induced re-location of IPS-1, generating mitochondrion (red staining) partially devoid of IPS-1 (green). (TIF) [file ppat.1005444.s007.tif]

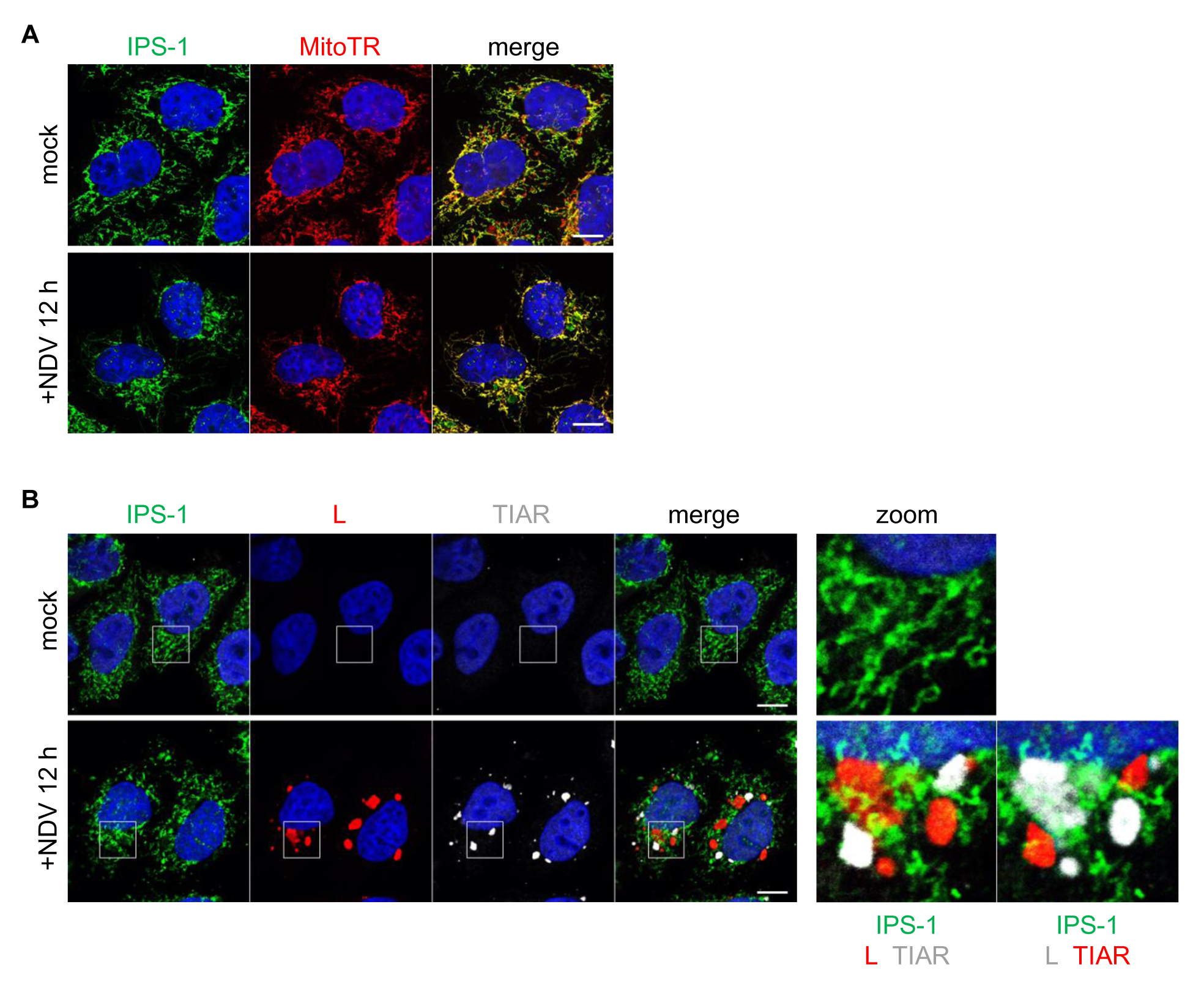

Supplement: S8 Fig — HeLa cells were either mock treated or infected with NDV (MOI = 1) for 12 hours. At 11.5 hpi, the medium was replaced with fresh medium containing 1 μM MitoTracker Red (A). The cells were then immunostained for IPS-1 (green, using a specific antibody produced in guinea pig), L (red), and TIAR (white). Nuclei were stained with DAPI (blue). The white scale bar corresponds to 10 μm. The boxed area was enlarged and displayed on the right (zoom). Partial co-localization between IPS-1 and L (vRC) or TIAR (avSG) is shown by displaying them in green and red, respectively (zoom). (TIF) [file ppat.1005444.s008.tif]

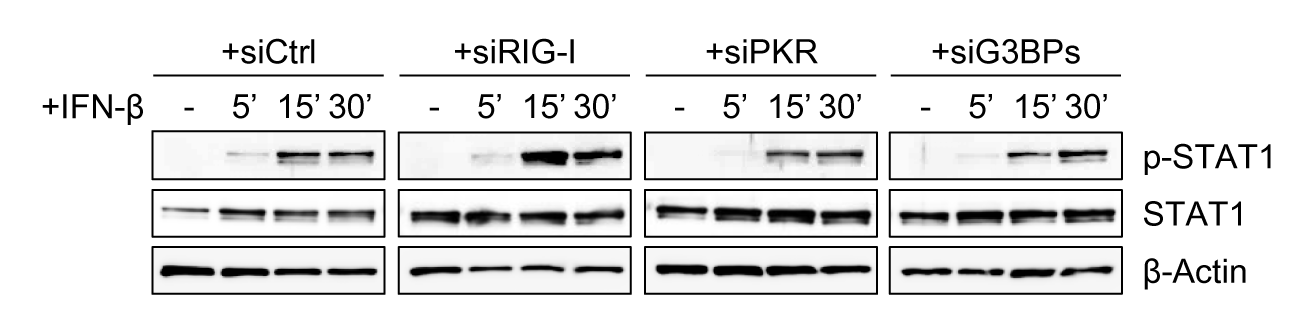

Supplement: S9 Fig — HeLa cells were transfected with siRNAs; siCtrl, siRIG-I, siPKR, or siG3BPs (for G3BP1 and G3BP2) for 48 hours. The cells were then stimulated with IFN-β (1,000 U/ml) for the indicated time. Phosphorylation level of STAT1 was detected by western blotting. (TIF) [file ppat.1005444.s009.tif]

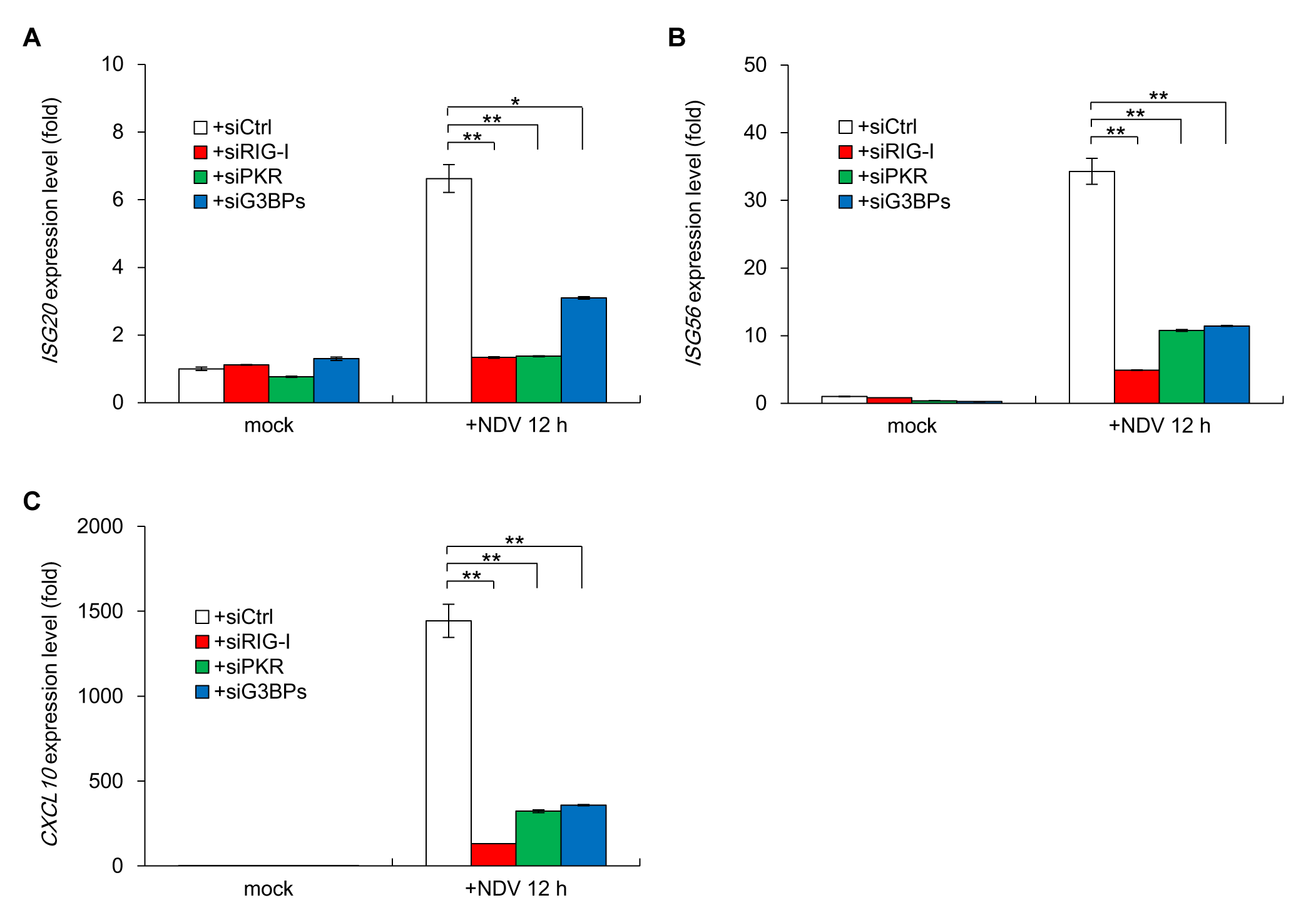

Supplement: S10 Fig — HeLa cells were transfected with siRNAs; siCtrl, siRIG-I, siPKR, or siG3BPs (siG3BP1 and siG3BP2). After transfection, the cells were mock treated or infected with NDV (MOI = 1) for 12 hours. Expression levels of ISG20, ISG56, and CXCL10 mRNA were measured by qRT-PCR. Data are represented as means ±SD (t-test: ***p<0.01, **p<0.05, *p<0.1, NS = not significant). (TIF) [file ppat.1005444.s010.tif]

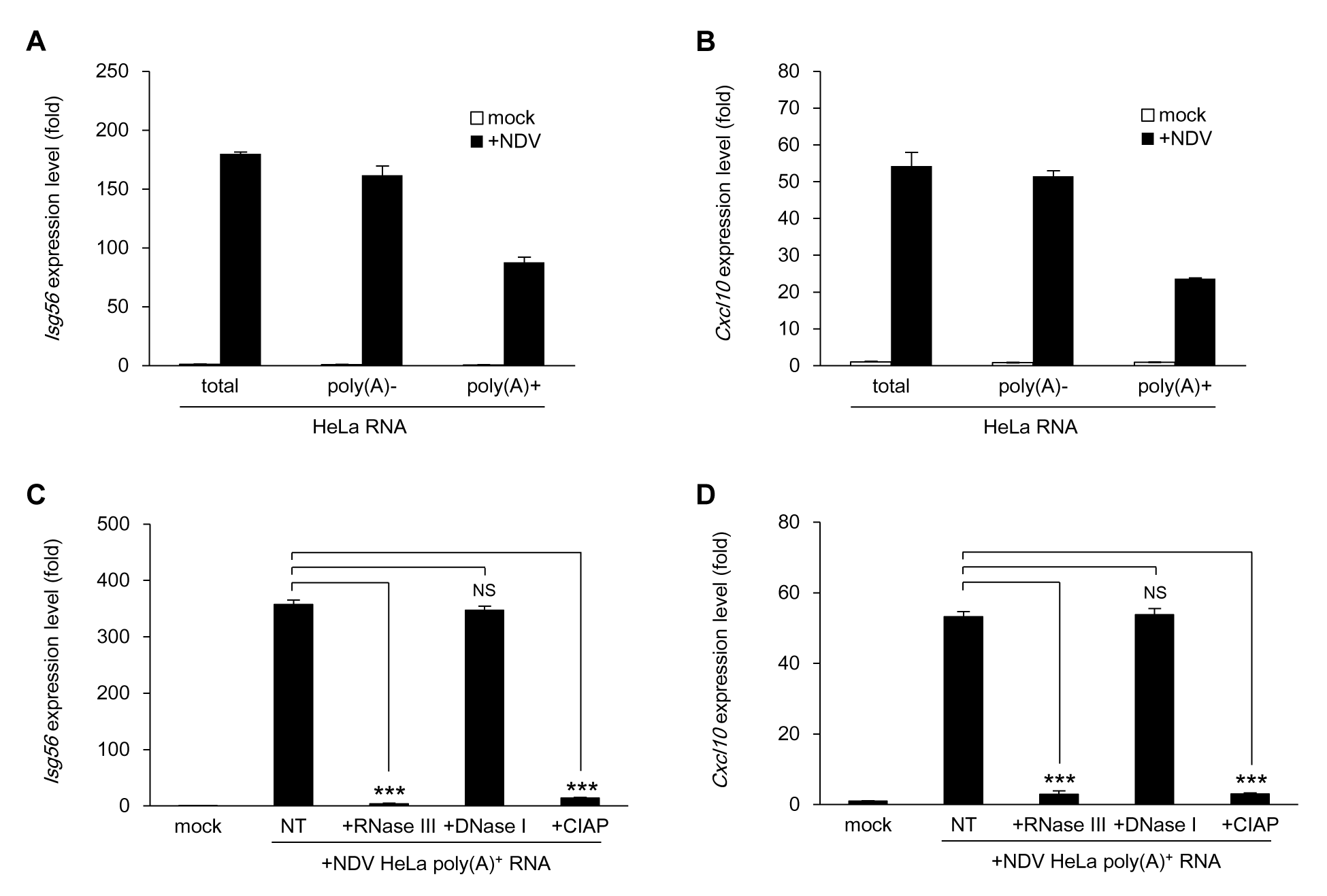

Supplement: S11 Fig — (A and B) MEFs (2×10^5^ cells) were transfected with total, poly(A)-, or poly(A)+ RNA (200 ng) from mock/NDV-infected (MOI = 1) HeLa cells. Isg56 and Cxcl10 mRNA expression levels were measured by RT-qPCR. (C and D) Poly(A)+ RNA (200 ng) from NDV-infected (MOI = 1) HeLa cells was mock treated (NT) or treated with RNase III, DNase I, or CIAP, and then transfected to MEFs (2×10^5^ cells). Isg56 and Cxcl10 mRNA expression was quantified by RT-qPCR. Data are represented as means ±SD. (TIF) [file ppat.1005444.s011.tif]

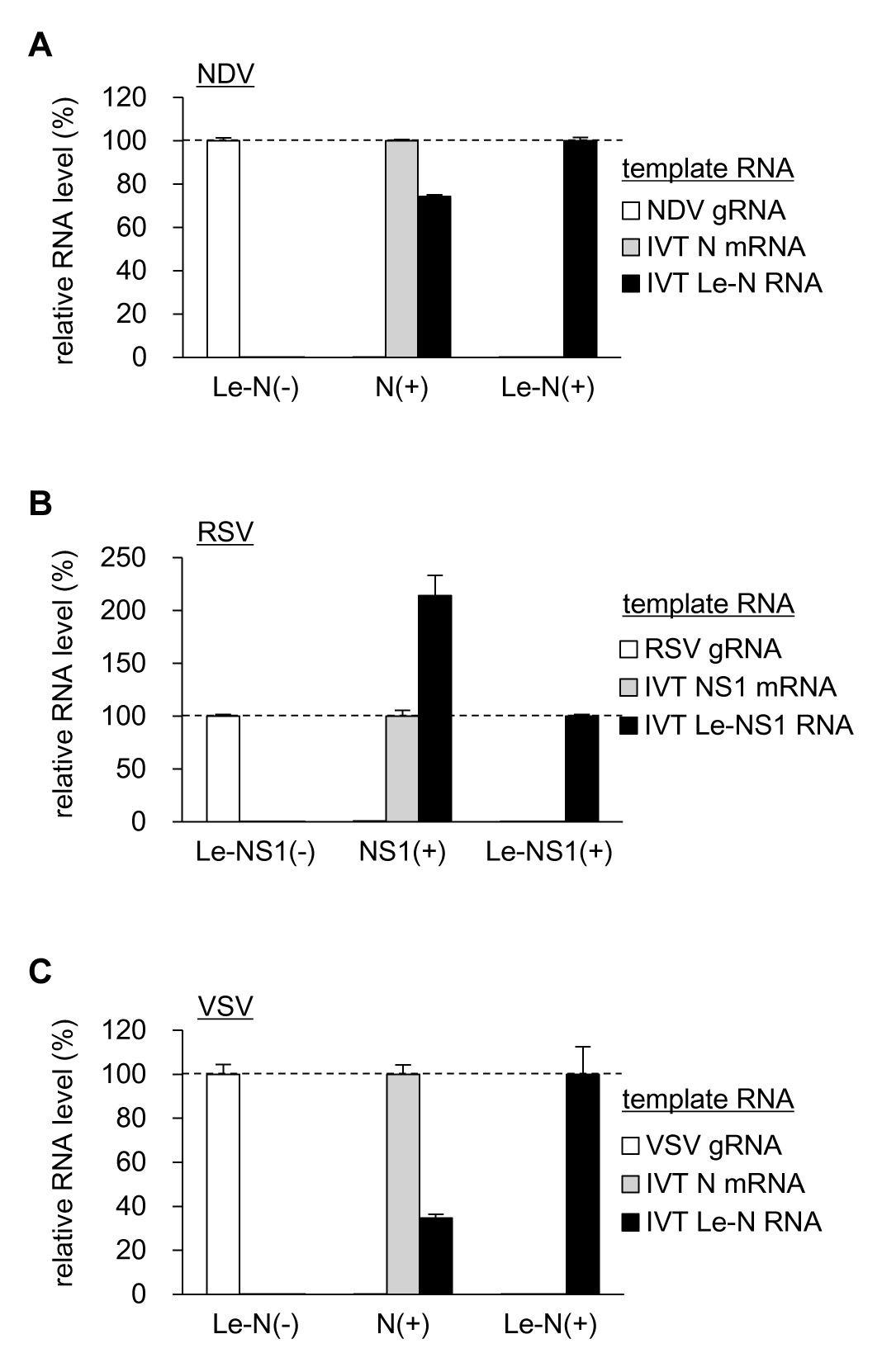

Supplement: S12 Fig — (A-C) 1010 copies of vgRNA isolated from the viral particles and in vitro synthesized RNA corresponding to vmRNA (N, NS1 and N for NDV, RSV and VSV, respectively) and read-through RNA (Le-N, Le-NS1 and Le-N for NDV, RSV and VSV, respectively) were subjected to strand-specific RT-qPCR (ssRT-qPCR) using specific primer sets (Tables in S2, S3 and S4 Tables). Percentage of the RNA copies of each target RNA was shown. Data are represented as means ±SD. The results showed specificity of ssRT-qPCR: probe for vRNA(-) only detected vgRNA; probe for Le-N/NS1(+) read-through RNA selectively detected Le-N/NS1 RNA but not N mRNA. (TIF) [file ppat.1005444.s012.tif]

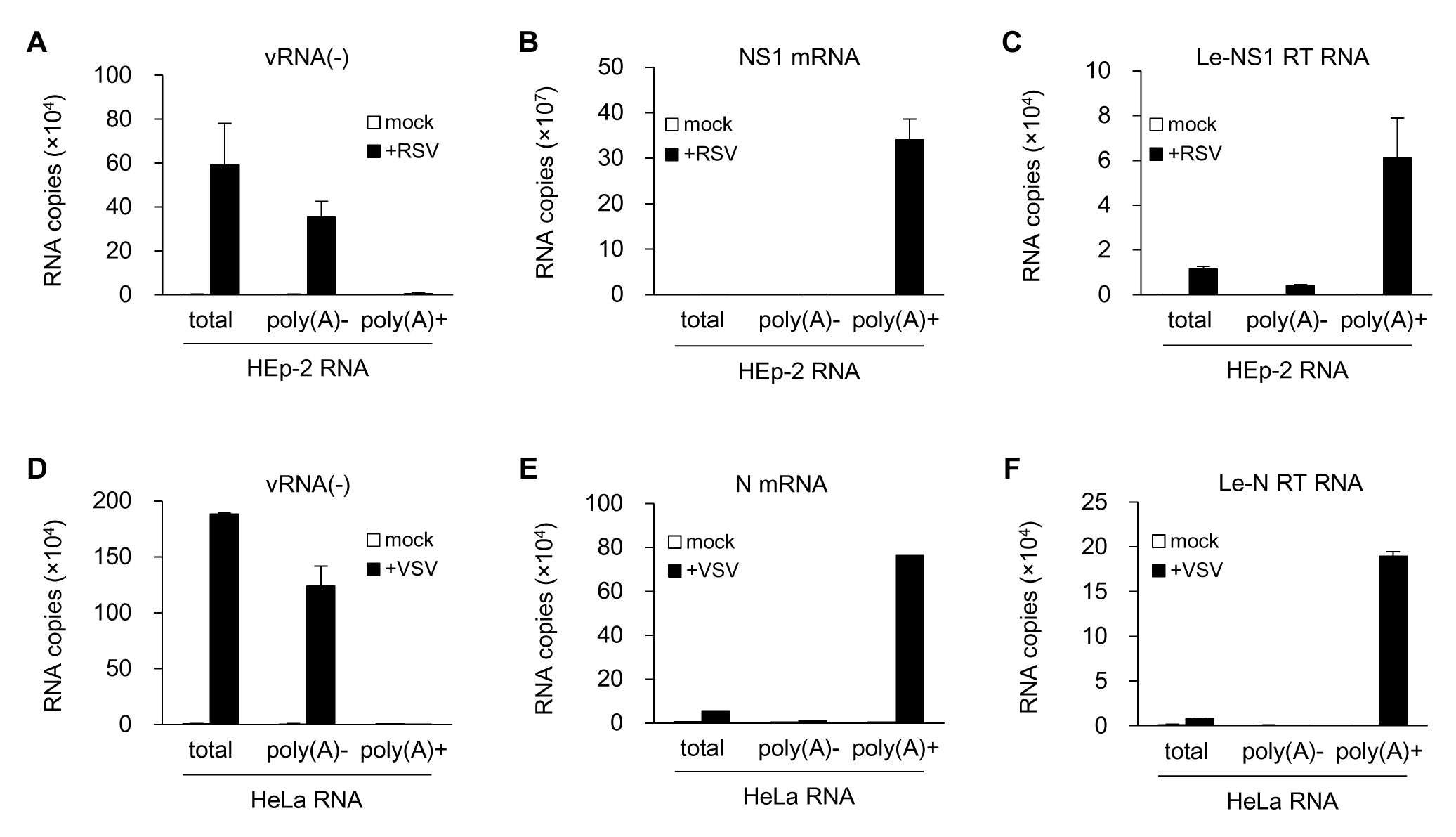

Supplement: S13 Fig — (A-F) Total, poly(A)-, and poly(A)+ RNA from mock treated, RSV-infected (60 hpi, MOI = 1) HEp-2 cells (A-C), or VSV-infected (12 hpi, MOI = 1) HeLa cells (D-F) were subjected to strand-specific RT-qPCR (ssRT-qPCR) targeting Le-NS1/N(-) as a portion of vgRNA, NS1/N(+) vmRNA, and Le-NS1/N(+) read-through RNA with specific primer sets (Tables in S3 and S4 Tables). Data are represented as means ±SD. (TIF) [file ppat.1005444.s013.tif]

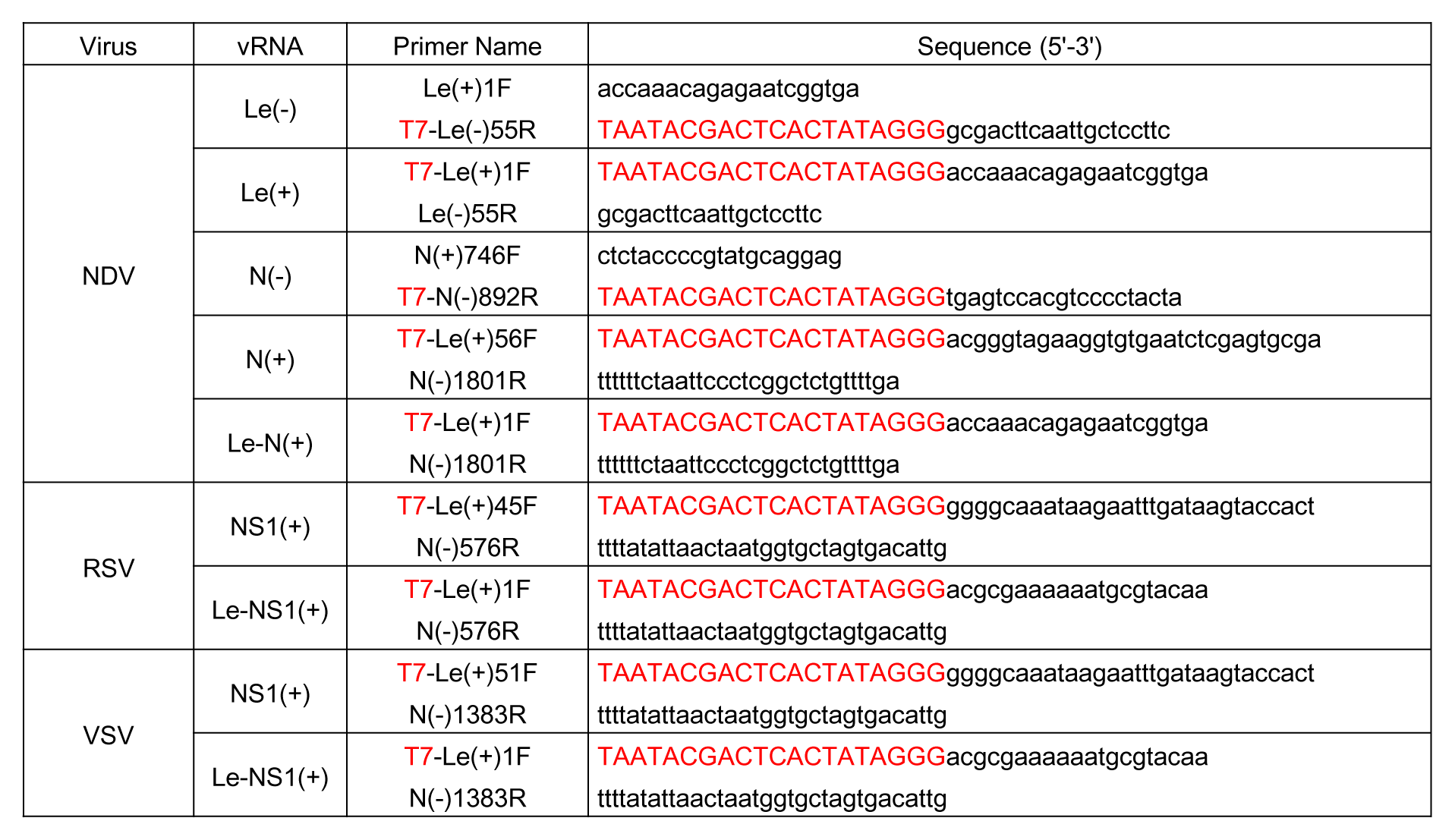

Supplement: S1 Table — (TIF) [file ppat.1005444.s014.tif]

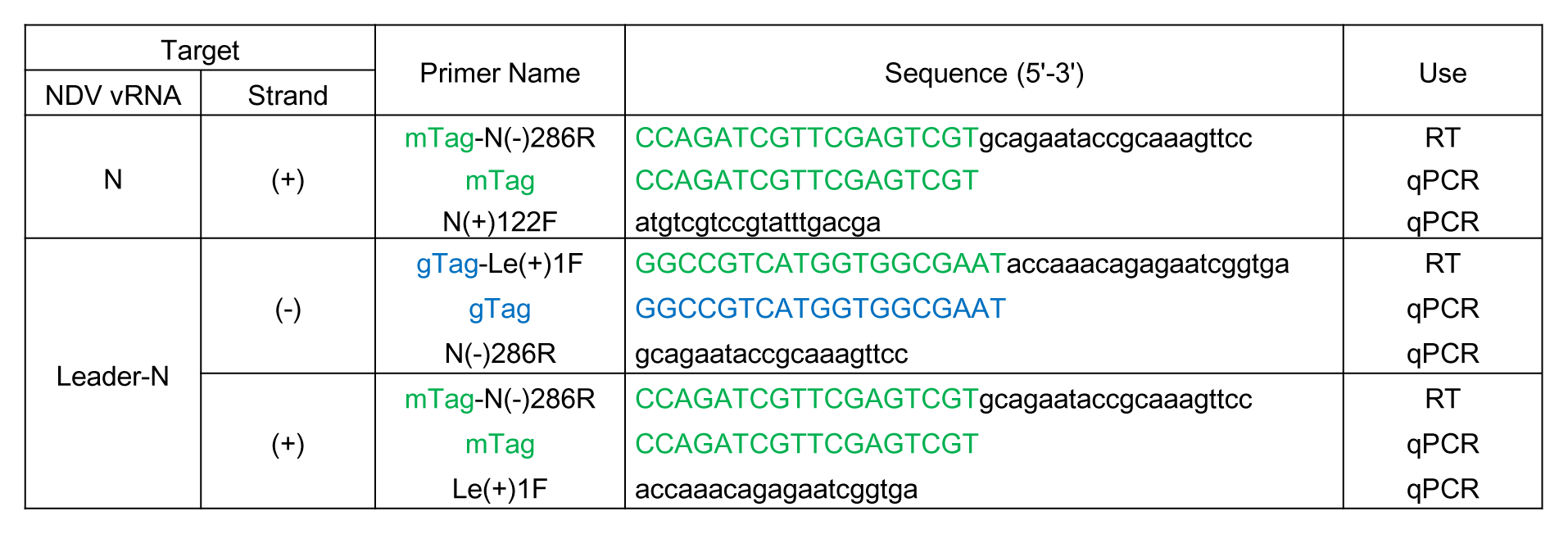

Supplement: S2 Table — (TIF) [file ppat.1005444.s015.tif]

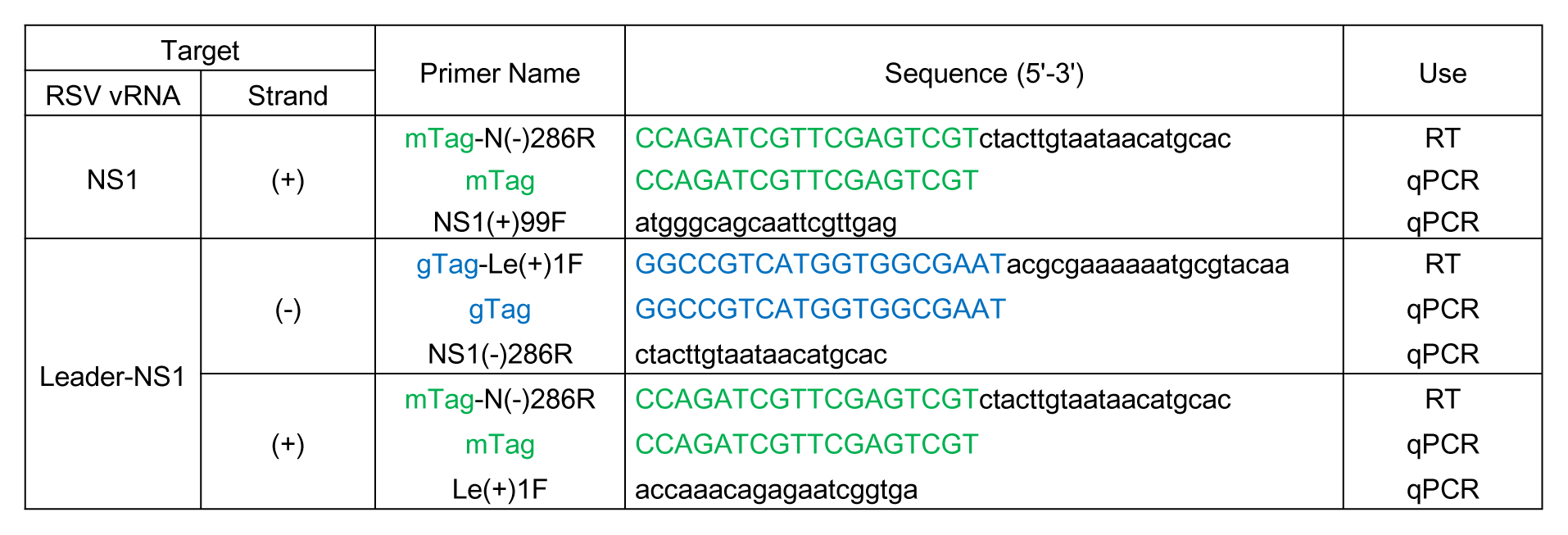

Supplement: S3 Table — (TIF) [file ppat.1005444.s016.tif]

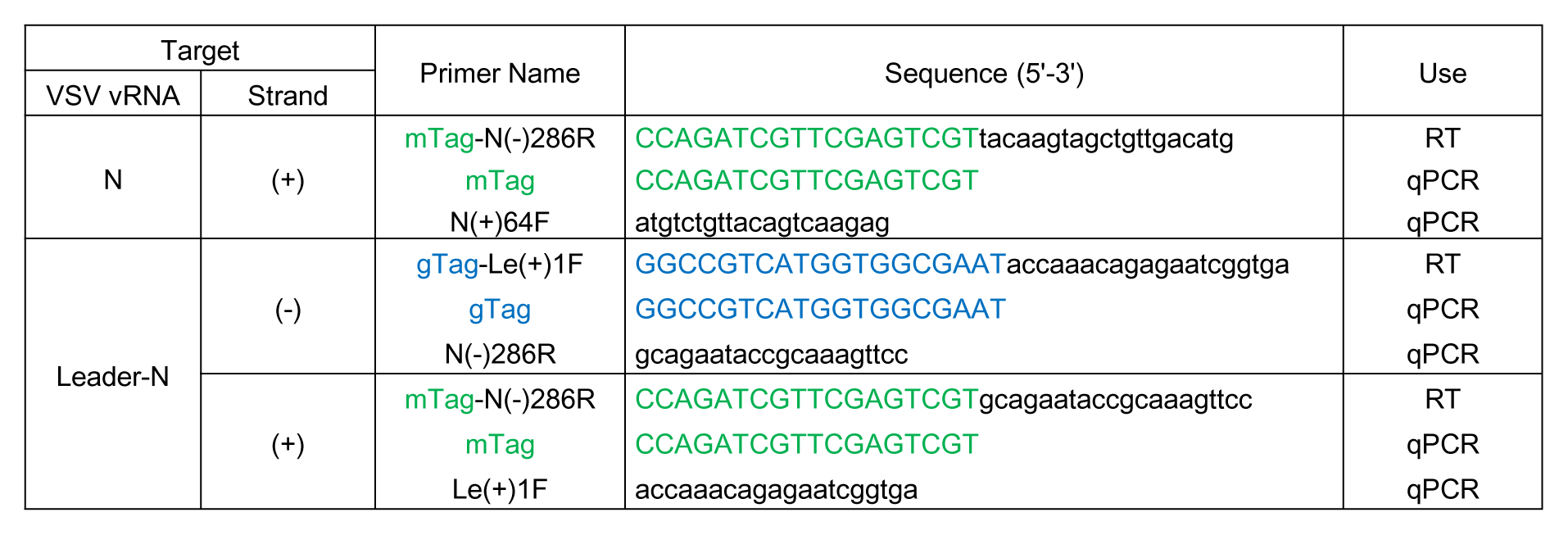

Supplement: S4 Table — (TIF) [file ppat.1005444.s017.tif]
